# Cutaneous Leishmaniasis and Sand Fly Fluctuations Are Associated with El Niño in Panamá

**DOI:** 10.1371/journal.pntd.0003210

**Published:** 2014-10-02

**Authors:** Luis Fernando Chaves, José E. Calzada, Anayansí Valderrama, Azael Saldaña

**Affiliations:** 1 Institute of Tropical Medicine (NEKKEN), Nagasaki University, Sakamoto, Nagasaki, Japan; 2 Programa de Investigación en Enfermedades Tropicales (PIET), Escuela de Medicina Veterinaria, Universidad Nacional, Heredia, Costa Rica; 3 Instituto Conmemorativo Gorgas de Estudios de la Salud (ICGES), Ciudad de Panamá, República de Panamá; Liverpool School of Tropical Medicine, United Kingdom

## Abstract

**Background:**

Cutaneous Leishmaniasis (CL) is a neglected tropical vector-borne disease. Sand fly vectors (SF) and *Leishmania* spp parasites are sensitive to changes in weather conditions, rendering disease transmission susceptible to changes in local and global scale climatic patterns. Nevertheless, it is unclear how SF abundance is impacted by El Niño Southern Oscillation (ENSO) and how these changes might relate to changes in CL transmission.

**Methodology and Findings:**

We studied association patterns between monthly time series, from January 2000 to December 2010, of: CL cases, rainfall and temperature from Panamá, and an ENSO index. We employed autoregressive models and cross wavelet coherence, to quantify the seasonal and interannual impact of local climate and ENSO on CL dynamics. We employed Poisson Rate Generalized Linear Mixed Models to study SF abundance patterns across ENSO phases, seasons and eco-epidemiological settings, employing records from 640 night-trap sampling collections spanning 2000–2011. We found that ENSO, rainfall and temperature were associated with CL cycles at interannual scales, while seasonal patterns were mainly associated with rainfall and temperature. Sand fly (SF) vector abundance, on average, decreased during the hot and cold ENSO phases, when compared with the normal ENSO phase, yet variability in vector abundance was largest during the cold ENSO phase. Our results showed a three month lagged association between SF vector abundance and CL cases.

**Conclusion:**

Association patterns of CL with ENSO and local climatic factors in Panamá indicate that interannual CL cycles might be driven by ENSO, while the CL seasonality was mainly associated with temperature and rainfall variability. CL cases and SF abundance were associated in a fashion suggesting that sudden extraordinary changes in vector abundance might increase the potential for CL epidemic outbreaks, given that CL epidemics occur during the cold ENSO phase, a time when SF abundance shows its highest fluctuations.

## Author Summary

In this study we analyze data on sand fly (SF) abundance and cutaneous leishmaniasis (CL) cases from Panamá. We asked whether weather patterns and climatic variability could have an impact on vector abundance that is ultimately reflected in CL transmission. We found that large epidemics of CL occur during the cold phase of El Niño Southern Oscillation (ENSO), with regular cycles coinciding with the oscillation patterns of ENSO. By contrast, and counterintuitively, we found that during the hot and cold phase of ENSO the average number of SF was reduced when compared with the normal phase of ENSO. Nevertheless, the cold ENSO phase also shows the largest variability in vector abundance, which is a likely indicative of sudden and extraordinary increases in SF abundance. We, therefore, propose that marked SF abundance changes, triggered by anomalous weather patterns associated with ENSO, likely play a major role in shaping CL interannual transmission cycles in Panamá.

## Introduction

Cutaneous leishmaniasis (CL) is a major neglected tropical disease [1] with a complex ecology [2], whose transmission, in the New World, requires the co-existence of vectors, reservoirs and humans [3], [4]. In Panamá, detailed studies on the eco-epidemiology of the disease [2], [5] described parasitological aspects of reservoirs [6] and vectors [7] and the environmental context of parasite-reservoir-vector interactions [8]. These studies set several landmarks for understanding New World CL epidemiology, including the demonstration of two toed sloths, *Choloepus hoffmanni* [9], [10] and other mammals [11], [12] as reservoirs of *Leishmania* spp parasites. Insights on sand fly vector ecology included: catholic bloodfeeding patterns in dominant vector species [13], [14], very limited dispersal of flying adults [15], high sensitivity of larval biology to environmental factors [16], [17], [18], [19], [20], differential adult resting behavior with species segregating along tree height [21], large diversity of co-occurring sand fly species across CL transmission foci [22], [23], [24] and heterogeneities in species composition across landscape gradients [23]. Currently, the most common parasite causing CL in Panamá is *Leishmania panamensis* [25], [26] and the resurgence and exacerbation of disease transmission has led to renewed efforts aimed at improving vector control [27] as a measure to reduce transmission at emerging transmission hotspots [25]. However, larger questions about what is driving the resurgence of the disease, and how to best predict epidemic outbreaks remain unanswered [28], [29].

The longitudinal nature of eco-epidemiological studies on CL in Panamá [2] revealed interannual patterns of variability in reservoir infection and abundance [10] and the sensitivity of sand fly density to weather fluctuations [30]. Nevertheless, no study has addressed if large scale meteorological phenomena, such as El Niño Southern Oscillation (ENSO), are associated with interannual CL epidemic cycles, as observed in neighboring Costa Rica [28], nor the impacts of ENSO on Sand Fly populations. These issues are of special interest, given the increased reports of direct and indirect impacts of climatic variability patterns associated with global warming on the disease transmission, both in the New World [32], [33], [34], [35], [36], [37], [38] and the Old World [39], [40]. Here, we employ an 11 years long (2000–2010) monthly time series recording CL cases in the República de Panamá to investigate seasonal and interannual cycles on this disease. During this time at the coarse spatial scale forest cover has marginally increased, yet locally some areas have seen deforestation [41], an activity usually linked with CL outbreaks [42]. We specifically ask which climatic factors are associated with seasonal and interannual disease cycles, considering local temperature and rainfall records and sea surface temperature 4 (SST4), an index associated with ENSO activity in the region. We also ask whether dominant sand fly (SF) vector species undergo marked abundance changes associated with ENSO, that are ultimately reflected in CL transmission. We employed seasonal autoregressive models and cross wavelet coherence analysis to depict the association of CL with climatic factors, and found that while seasonal CL patterns were mainly associated with temperature and rainfall, interannual cycles of the disease were associated with SST4. Moreover, SST4 was also associated with interannual cycles in temperature and rainfall. SF vectors showed marked abundance changes associated with ENSO, where abundance in general decreased during the hot and cold phases of ENSO. Our results highlight both the general association of ENSO and weather patterns with CL dynamics in Central America, and how changes in SF vector abundance associated with ENSO might play a role in the emergence of CL epidemics.

## Materials and Methods

### Data

**Figure 1:**
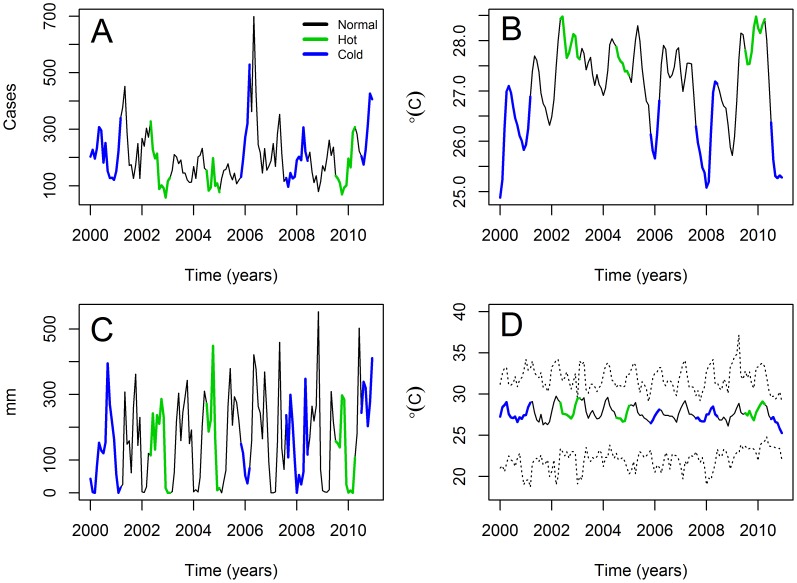
Monthly time series data (2000–2010). (A) Cutaneous Leishmaniasis cases in the Republic of Panamá (B) Rainfall (C) Temperature. The solid line indicates the averages and dashed lines the extremes. (D) Sea Surface Temperature 4 (El Niño 4 Index). All time series start in January 2000 and end in December 2010. In the plots colors indicate the ENSO phase, for details refer to the inset legend in panel A.

Monthly CL cases were compiled by the Epidemiology Department of Panamá's Ministry of Health for the period January 2000 – December 2010. Briefly data consisted of cases clinically diagnosed [43], and often confirmed by the microscopic examination of skin lesson scrappings/biopsies, Montenegro skin tests (MST) [44] or Indirect Immuno-Fluorescent Agglutination Tests (IFAT) [26]. Data (Figure 1A) were collected from all the health facilities administered by Panamá's Ministry of Health and all data came from passive case detection. Reports were then compiled at the health area level (the operational geographical units of Panamá's Ministry of Health which are slightly different from Panamá's provinces and autonomous indigenous comarcas). Slightly over 80% of the cases came from West Atlantic Panamá, the area facing the Caribbean Sea, west of the Panamá Canal up to the border with Costa Rica [25]. Representative samples from all over Panamá [25] indicate that over 95% (at least 90% for each reporting health area) of the CL cases in the time series are due to *Leishmania panamensis*. Cases due to *Leishmania mexicana*, *Le amazonensis* and *Le colombiensis* continue to be rare and sporadic, as observed in earlier epidemiological studies in Panamá [5]. Moreover, all CL cases observed in migrants that likely acquired the infection in Panamá have been typified as *Le panamensis* [45], [46]. Temperature data were obtained from the US National Oceanic and Atmospheric Administration, NOAA (ftp://ftp.ncdc.noaa.gov/pub/data/ghcn/v2/) for Tocumen (Station 787920), Albrook (Station 783842 and 788060), Bocas del Toro (Station 787935), David (Station 787930) and Santiago (Station 787950). These daily time series were averaged per month, considering all values and the monthly maxima and minima (Figure 1B). Rainfall data, an average considering all weather stations, were obtained from ETESA, Panamá's electrical company (Figure 1C). Monthly SST 4, often referred as El Niño 4 (Figure 1D), was obtained from (http://www.cpc.ncep.noaa.gov/data/indices/ersst3b.nino.mth.81-10.ascii). The NOAA data for SST4 were collected from the area delimited by 5°North-5°South and 160°East-150°West of the Pacific Ocean. We also classified each month from the time series into the ENSO phases following the Oceanic Niño Index (ONI) from NOAA (http://www.cpc.ncep.noaa.gov/products/analysis_monitoring/ensostuff/ensoyears.shtml). Briefly, the ONI is estimated by detrending the monthly Sea Surface Temperature 3.4 (collected in the area defined by 5°N-5°S, 120°-170°W) over periods of 30 years, with residues with a value over 0.5 corresponding to the warm (or hot) ENSO phase, residues below −0.5 corresponding to the cold ENSO phase and residues in the interval [−0.5,0.5] being normal conditions [47].

SF abundance data came from six studies performed either at Universidad de Panamá or Instituto Conmemorativo Gorgas de Estudios de la Salud (ICGES). All these studies were performed within the República de Panamá (Figure S1A). All the studies used a common sand fly sampling method, which consisted in the use of unbaited light traps placed at 1.5–2.0 m height above ground, with traps operating from 6 pm to 6 am (a sampling effort referred as trap-night), and placed in three well defined eco-epidemiological environments: (i) domiciliary for samples from inside houses; (ii) peridomiciliary for samples collected in a radius of 100 m from a house; and (iii) forested areas for samples collected in areas with primary/secondary vegetation and outside a 100 m radius from a house. This standardized sampling, in principle, renders the comparison of the different datasets plausible. In most of the studies sand fly sampling was done with the purpose of describing the fauna at endemic locations [48], [49], [50], as part of the evaluation of SF control trials [27] and we also report unpublished data from entomological surveillance of endemic CL locations in 2007, 2009, 2010 and 2011. We focused our analysis in the abundance of *Lutzomyia trapidoi*, *Lu gomezi* and *Lu panamensis* (Figure S1B), the dominant vector species for CL in the República de Panamá [2], [7]. This was done given the proven vectorial role of these species [2], [7], and the lack of abundant records for other sand fly species. Figure S1C shows the eco-epidemiological environments sampled in each location and Figure S1D the year when samples were collected at each location. A total of 12580 SF were collected over 640 trap-nights. In all the studies, the spermathecae or genitalia were inspected for species identification using the key by Young and Duncan [51].

### Statistical Analysis

#### Leishmaniasis seasonality, interannual cycles and non-stationary associations with weather records and ENSO

Seasonality in the CL cases time series was assessed with monthly boxplots [52] and seasonal time series plot depicting the ENSO phase. The correlation structure in the CL time series was assessed by inspecting both the autocorrelation function, ACF, i.e., the time series correlation with itself through time, and the partial autocorrelation function, PACF, i.e., the correlation between consecutive time lags [53]. Information from the ACF and PACF was used to choose the time lags necessary to fit a null model, i.e., without climatic covariates, of the CL time series. The null model coefficients were used to pre-whiten the climatic covariates SST4, Rain, TMAX and TMIN. Pre-whitening is a process that removes the common autoregressive structure of an ancillary time series (often referred as filtering) thus easing the study of association patterns with a focal time series [53]. Null model residuals together with pre-whitened residuals from the climatic time series were then used to estimate cross-correlation functions, CCF, of CL with each one of the climatic covariates. This information was employed to build a full model that was simplified to avoid having more parameters than what is necessary to understand the dynamics of the CL time series, i.e., to avoid over-parameterization [54]. We used backward elimination of climatic covariates for model simplification, i.e., taking the least significant covariate by rounds [53]. We used the Akaike Information Criterion, AIC, to choose the best model in each round of backward elimination. AIC is a model selection criterion that picks the best model, in a group, once a trade-off function between the number of parameters and likelihood is minimized [53]. Finally, in all cases, assumptions about model error, normality and independence, were verified using standard procedures for time series analysis [53]. To better understand the process of model association we studied the correlation of the variables considered in the full model by estimating the correlation between all variables pairs and plotting the correlations in color and size categories according to their and magnitude, building a corrgram [55]. Finally, time-frequency association of CL with climatic covariates was studied using continuous wavelet transforms [56] to estimate a cross-wavelet coherence. This analysis depicts the nonstationary association between time series [56], [57], i.e., their non-constant association through time, specially by depicting the coherence, i.e., association of cycles between two time series over time [28].

#### Sand fly vector abundance and ENSO

We fitted a Poisson Rate Generalized Linear Mixed Model (PRGLMM) [58] to abundance records from each of the dominant SF vector species (*Lu gomezi, Lu trapidoi* and *Lu panamensis*). We chose PRGLMMs given the counting nature of our data [58] and to consider the lack of independence that emerges from observations collected across different studies [59]. The model was a rate model given that for a couple of studies [30], [49] we had records that came from several traps and, therefore, for parameter estimation we needed to include an offset variable to account for the heterogeneous number of traps generating the counts [60]. In the models we considered the following fixed factors: (a) ENSO phase, to quantify patterns of interannual variability in SF abundance; (b) sampling month, to account for seasonal changes in SF abundance and (c) the sampling eco-epidemiological environment, to account for variability that can be attributed. We considered as random factors the variability that can be attributed to: (d) each study and the nested variability that can be attributed to each location within each study in order to consider the spatial variability that emerges from observations across different locations; (e) the temporal variability that could have emerged from samples collected in different years. For the analyses, we only considered SF abundance from traps used in the control treatment of the SF vector control trial study [27], i.e., records from houses no subjected to any pesticide, while we used data from all the light traps used in the other studies [47], [48], [49] and from unpublished vector surveillance records.

#### Sand fly vector abundance and cutaneous leishmaniasis cases

To study the relationship between CL incidence and SF vector abundance, we employed the SF trap records employed to study the relationship between SF abundance and ENSO and estimated average numbers of SF/trap-night/month. We were able to obtain 35 *Lu gomezi* monthly abundance estimates (MAES), 36 *Lu trapidoi* MAES and 37 *Lu panamensis* MAES, in the period January 2000 – December 2010. We employed this data together with data from the CL time series to study their cross-correlation patterns [53]. From our previous mathematical modelling efforts we expected a positive relationship between the number of cases and vector abundance [4], [61], [62], [63], [64], with the abundance of vectors leading the number of cases with a time lag [29].

## Results

**Figure 2:**
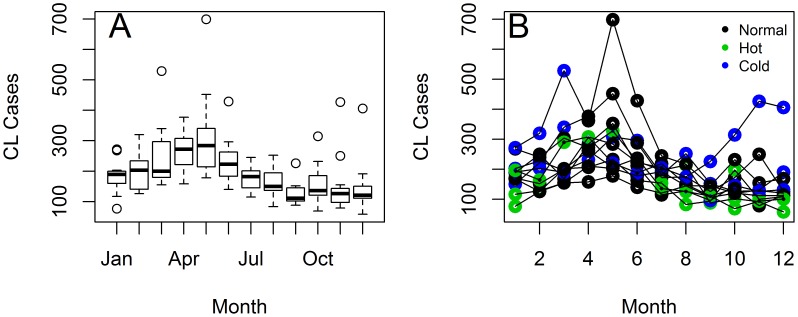
Cutaneous Leishmaniasis cases (CL) seasonality. (A) Boxplots of monthly incidence. Boxes contain data within the 25^th^ to 75^th^ quantiles. Lines inside the boxes show the median of the distribution for each month. (B) Seasonal (year-long) time series. Colors indicate the ENSO phase, see inset legend for details.

Figure 2 shows CL case seasonality. Figure 2A shows how CL cases peak at the beginning of the rainy season in Panamá, in April and May [41]. From January 2000 to December 2010 a total of 26140 CL cases were recorded in Panamá. Figure 2B shows how ENSO phases have been uniformly distributed across the year, and how peaks in CL cases tend to occur during the cold ENSO phase, a pattern also shown in Figure S2 when pooling all months. Given the monthly nature of our data and the focus on the total number of reported CL cases, with observations spanning 132 months, we employed a battery of tools for time series analysis in the time domain, including temporal correlation functions and seasonal autoregressive models, and time-frequency domain, i.e., wavelets [65]. Figure S3 show the different correlation functions employed to fit seasonal autoregressive models to the CL time series. The PACF (Figure S3A) suggested a seasonal autoregressive structure in the CL time series, where up to the first three lags and the seasonal lag (12 months) were associated with CL number at any time. The autoregressive pattern was also suggested by the ACF (Figure S4). Indeed a 3^rd^ order seasonal autoregressive model was selected as the best null model (Table S1) and employed for pre-whitening climatic covariates and for subsequent estimation of Cross Correlation Functions (CCFs) between CL and climatic covariates (Figure S3B, S3C, S3D, S3E, S3F). CL was negatively correlated with SST4 at lags 4 and 5 and positively correlated with temperature at lag 12 (Figure S3B). CL was negatively correlated with rainfall (Rain) at lag 15 (Figure S3C). CL was positively correlated with average (Temp) and maximum temperature at lag 13 (Figure S3D & S3E) autonomous from minimum temperature (Figure S3F). Thus, based on the significant correlations observed in Figure S3 the following null model was fitted:
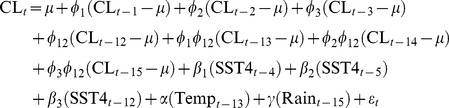
(1)


Where μ is the mean value of the time series, φ's indicate autoregressive parameters, β, γ and α are parameters for climatic covariates and ε indicates an error normally distributed and with variance 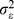. After several rounds of backward elimination (Table S1) the following model was selected as best:
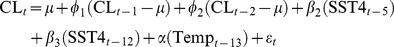
(2)


**Figure 3:**
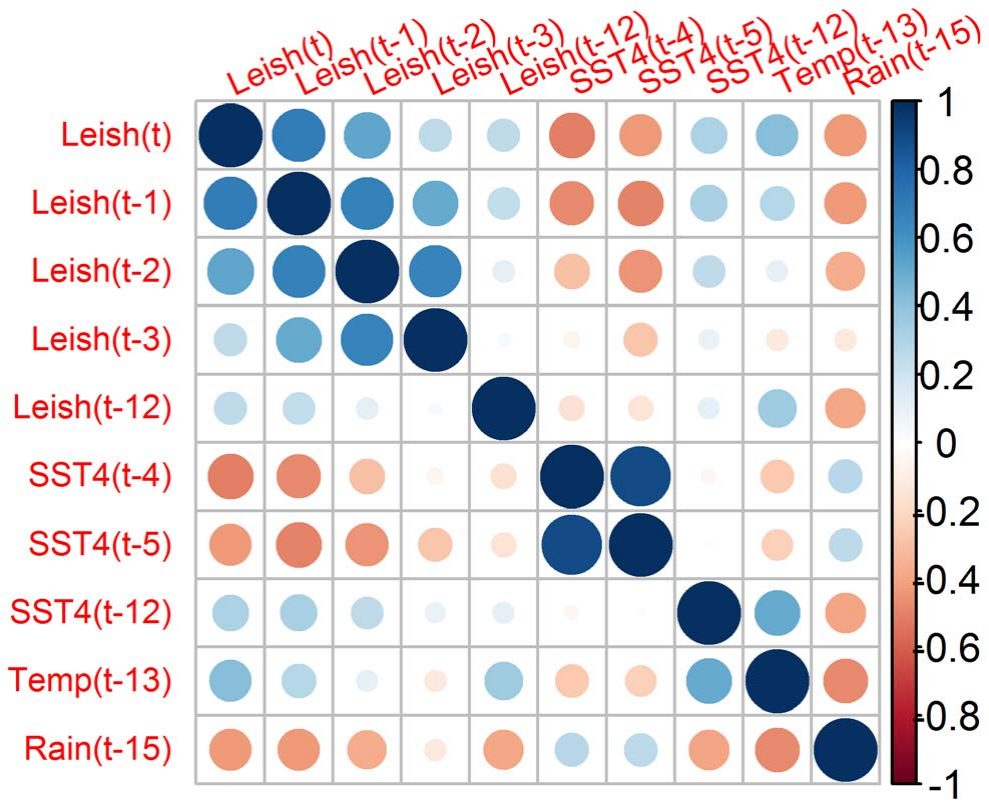
Correlation between selected lags of the Cutaneous Leishmaniasis cases from Republic of Panamá time series, and climatic covariates. Time lags are indicated inside parenthesis “()” and SST4, Temp and Rain are, respectively, abbreviations for Sea Surface Temperature 4 (El Niño 4 Index), Temperature and Rainfall. Circle size indicates the magnitude of the association, while color indicates the sign of the correlation, a scale is presented in the right margin of the figure.

Whose parameter estimates are presented in Table 1. The impacts of Temp(t-13) and SST4(t-12) on CL were positive, in contrast with SST4(t-5) which was negative, and assumptions about the error were met, ensuring a sound inference. The process of model selection (Table S1) left out variables that were strongly correlated (Figure 3) with the ones present in equation (2). For example, SST4(t-5), SST4(t-4) and Rain (t-15) were positively associated between them (Figure 3) and negatively with CL(t). Thus, SST4(t-5) was able to capture a common impact of ENSO on both rainfall and CL. Similarly, Temp(t-13) and CL(t-12) were positively associated between them and with CL(t) (Figure 3), rendering the inclusion of the former, in addition to SST4(t-5), enough to account for seasonality in CL(t).

**Table 1:** Parameter estimates for the best model.

Symbol	Parameter (Lag)	Estimate ± S.E.
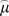	Intercept	210±23
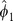	AR(1)	0.414±0.086
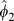	AR(2)	0.364±0.088
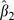	SST4(5)	−56.20±11.73
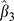	SST4(12)	33.82±13.21
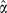	T(13)	32.96±8.42
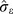	Error S.D.	57.18

AR, SST4 and T stand, respectively, for Autoregressive, Sea Surface Temperature 4 (El Niño 4 Index) and Temperature.

**Figure 4:**
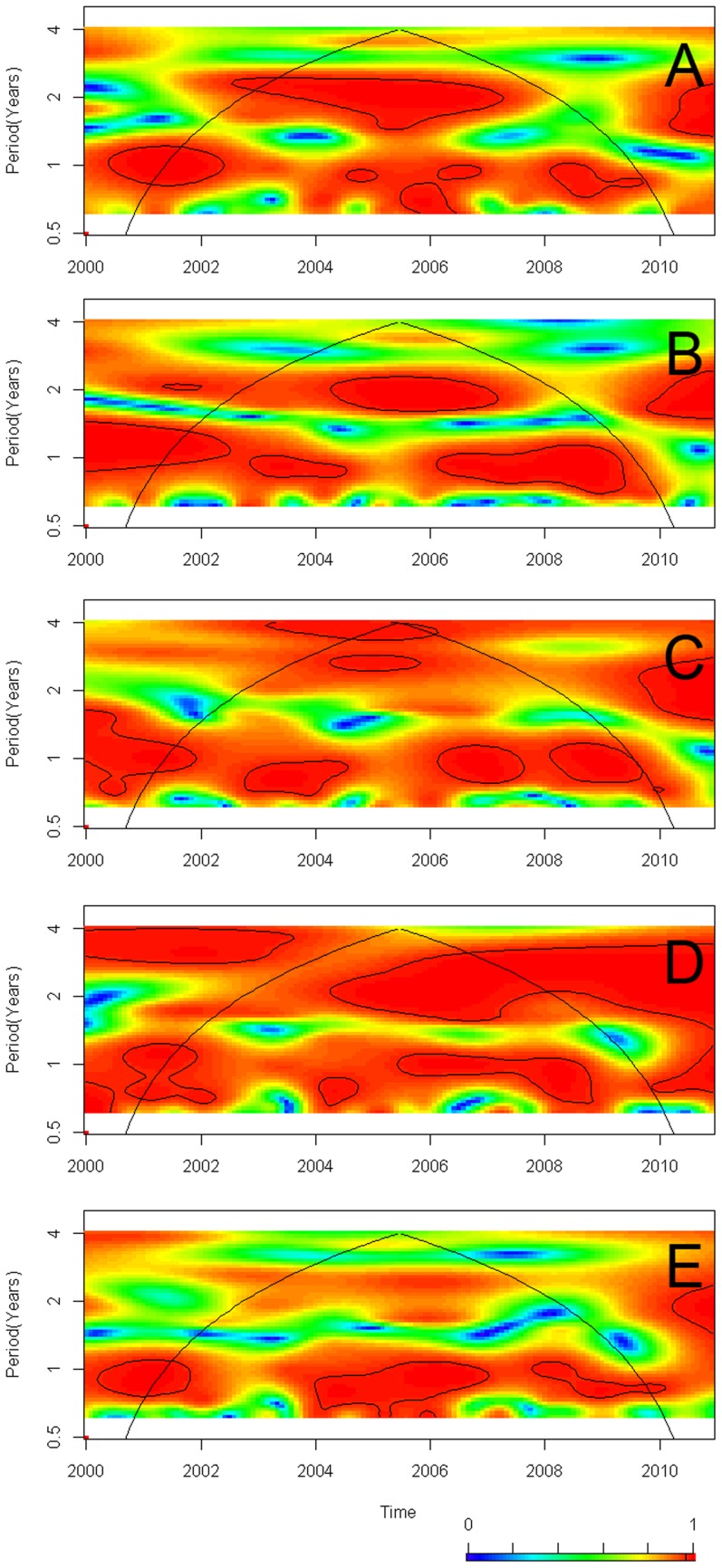
Cross-wavelet coherence analysis. Coherence between (A) Cutaneous Leishmaniasis cases in the Republic of Panamá, Leish, and Sea Surface Temperature 4, a.k.a., El Niño 4 index, SST4 (B) Leish and Rainfall, Rain (C) Leish and Average Temperature, Temp (D) Rain and SST4 (E) Temp and SST4. A cross wavelet coherence scale is presented at the bottom of the figure, which goes from zero (blue) to one (red). Red regions in the plots indicate frequencies and times for which the two series share power (i.e., variability). The cone of influence (within which results are not influenced by the edges of the data) and the significant coherent time-frequency regions (p<0.05) are indicated by solid lines.

The cross wavelet coherence analysis (Figure 4) confirms the outcome of autoregressive models, showing that CL was associated with: SST4 (Figure 4A), rainfall (Figure 4B) and temperature (Figure 4C) during the study period, all the three variables associated with the inter-annual variability in CL, specifically cycles with period between 2–4 years, the latter two also associated with the seasonal cycles (period of 1 year). Similarly, rainfall (Figure 4D) was associated with SST4 at inter-annual scales; and both rainfall (Figure 4D) and temperature (Figure 4E) were seasonally associated with SST4. In synthesis, ENSO, measured through SST4, shows an imprint on CL transmission robustly revealed by both the autoregressive model and the cross wavelet coherence analysis, where SST4 also impacts Rainfall and Temperature, which are, as well, associated with CL.

**Figure 5:**
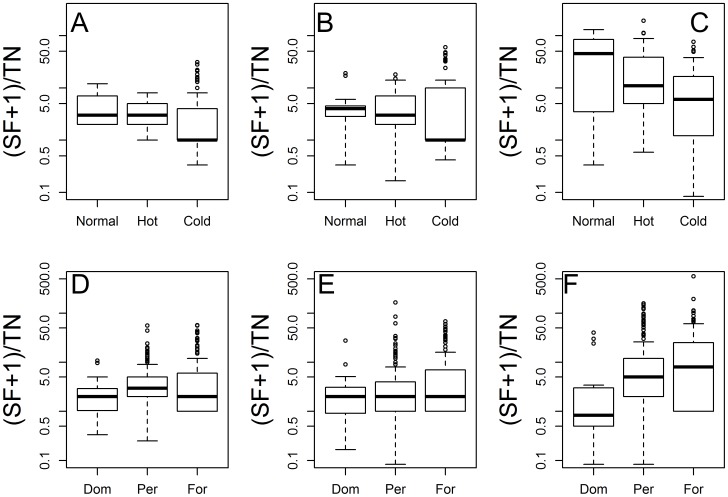
Sand Fly vector species abundance during the different ENSO phases and by eco-epidemiological environment. Abundance by ENSO phase: (A) *Lutzomyia gomezi* (B) *Lu trapidoi* (C) *Lu panamensis*. Abundance by eco-epidemiological environment: (D) *Lu gomezi* (E) *Lu trapidoi* (F) *Lu panamensis*. Panels A, B and C show data only for April, October and November where the number of trap-nights was above 30. In all panels the y-axis is in a logarithmic scale.

Data on SF vectors showed a common pattern where *Lu gomezi* (Figure 5A), *Lu trapidoi* (Figure 5B) and *Lu panamensis* (Figure 5C) reduced their average abundance during the hot and cold ENSO phases. Nevertheless, these three dominant SF vector species showed an increased variability in abundance during the cold ENSO phase, i.e., the boxes in the boxplots were longer, and large outliers were more frequent during the cold ENSO phase. After controlling for differences related to heterogeneity in eco-epidemiological sampling environment, and for seasonality associated with different sampling months, the GLMPMs for *Lu trapidoi* and *Lu panamensis* showed that abundance reductions were significant during both the hot and cold ENSO phases (P<0.05), but not for *Lu gomezi*, (Table 2). The abundance across eco-epidemiological environments showed a similar pattern for *Lu gomezi* (Figure 5D) and *Lu trapidoi* (Figure 5E), where abundance was slightly larger in domiciliary than in peridomiciliary or forest environments, a pattern statistically significant (Table 2). By contrast, *Lu panamensis* (Figure 5F) was, respectively, about 9 and 2.5 times more abundant in forests and peridomiciles than inside the houses (P<0.05, Table 2). Table 2 shows that, in general, the unexplained variance in the SF abundance GLMPMs associated with spatial heterogeneity (Location Var) was about one order of magnitude higher than the unexplained temporal variance (Year Var). Similarly, model selection with AIC and BIC showed that it was not necessary to nest the spatial random effects within each study (Table S2).

**Table 2:** Generalized linear mixed Poisson rate model parameter estimates.

Vector species	*Lutzomyia gomezi*					*Lutzomyia trapidoi*					*Lutzomyia panamensis*				
Parameter	PAC	Est	S.E.	z	Pr(>|z|)	PAC	Est	S.E.	z value	Pr(>|z|)	PAC	Est	S.E.	z	Pr(>|z|)
ENSO-Nomal, January and Domicile	1(3.21¶)	1.17	0.35	3.35	0.001*	1(3.86¶)	1.35	0.49	2.75	0.006*	1(2.17¶)	0.78	0.41	1.89	0.058
ENS0-Hot	0.81	−0.21	0.13	−1.63	0.103	0.25	−1.37	0.19	−7.38	1.55E-13*	0.21	−1.57	0.11	−14.12	<2e-16*
ENSO-Cold	0.95	−0.05	0.10	−0.49	0.622	0.60	−0.51	0.15	−3.52	<0.001*	0.15	−1.91	0.09	−22.28	<2e-16*
Peridomicile	0.55	−0.59	0.08	−7.58	3.57E-14*	0.31	−1.16	0.08	−13.78	<2e-16*	2.54	0.93	0.08	11.07	<2e-16*
Forest	0.48	−0.73	0.18	−4.11	4.03E-05*	0.47	−0.75	0.20	−3.81	<0.001*	9.96	2.30	0.11	21.53	<2e-16*
February	2.95	1.08	0.12	9.22	<2e-16*	8.78	2.17	0.13	17.17	<2e-16*	3.39	1.22	0.09	13.90	<2e-16*
March	1.56	0.45	0.12	3.86	<0.001*	0.71	−0.35	0.13	−2.62	0.009*	0.90	−0.10	0.10	−1.02	0.310
April	2.54	0.93	0.12	7.48	7.36E-14*	0.68	−0.38	0.17	−2.31	0.021*	0.75	−0.29	0.09	−3.05	0.002*
May	1.63	0.49	0.16	3.04	0.002*	0.21	−1.57	0.24	−6.62	3.51E-11*	0.45	−0.80	0.11	−7.47	7.78E-14*
June	2.62	0.97	0.16	5.95	2.71E-09*	0.41	−0.89	0.25	−3.61	<0.001*	0.53	−0.63	0.12	−5.11	3.23E-07*
July	0.69	−0.38	0.13	−2.97	0.003*	0.34	−1.07	0.17	−6.35	2.12E-10*	1.91	0.65	0.08	7.91	2.58E-15*
August	1.78	0.58	0.12	4.94	7.99E-07*	1.33	0.29	0.14	2.01	0.045*	3.13	1.14	0.08	14.69	<2e-16*
September	1.64	0.50	0.12	4.28	1.9E-05*	0.61	−0.49	0.16	−3.06	0.002*	1.99	0.69	0.09	8.09	6.11E-16*
October	1.15	0.14	0.12	1.12	0.263	1.40	0.34	0.15	2.31	0.021*	4.16	1.42	0.08	17.72	<2e-16*
November	2.04	0.72	0.17	4.28	1.87E-05*	4.60	1.53	0.18	8.45	<2e-16*	3.01	1.10	0.08	13.17	<2e-16*
December	0.39	−0.95	0.23	−4.03	5.64E-05*	1.27	0.24	0.17	1.40	0.162	1.43	0.36	0.10	3.51	<0.001*
Location Var		1.68					4.85					3.00			
Year Var		0.25					0.36					0.23			

PAC =  proportional abundance change, Est =  Estimate, Var =  Variance. Model assumptions were met, thus ensuring a sound inference.

*Statistically significant (P<0.05);

¶ Estimated Abundance/trap-night/month for domiciliary samples, collected in January during the normal phase of ENSO.

**Figure 6:**
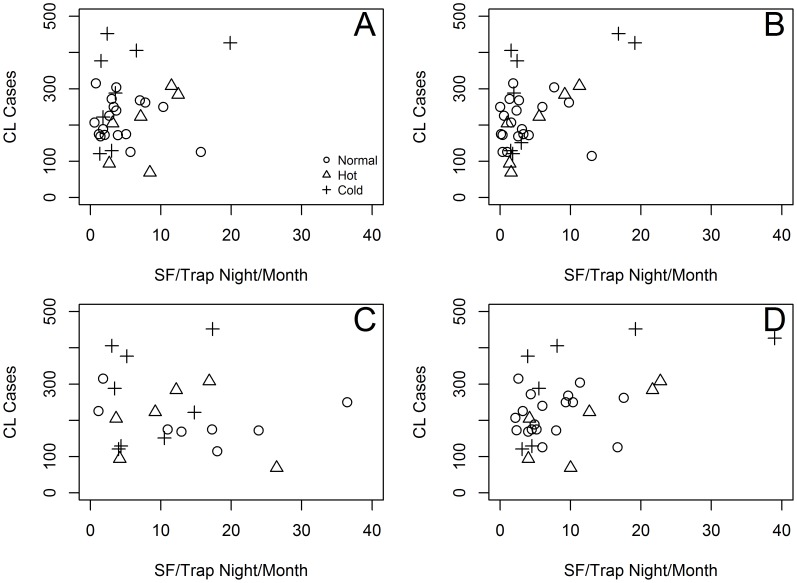
Cutaneous Leishmaniasis (CL) cases as function of dominant Sand Fly (SF) vector species abundance per trap-night and per month. (A) *Lutzomyia gomezi* (B) *Lu trapidoi* (C) *Lu panamensis* (D) *Lu gomezi* and *Lu trapidoi*. Panels show CL cases as function of SF abundance three months earlier.

Monthly CL case records were positively associated with the abundance of *Lu gomezi* (Figure 5SA) and *Lu trapidoi* (Figure 5SB) with the CCF peaking at three months lag, i.e., after a peak in SF vector abundance there was a peak in CL cases three months later. For *Lu gomezi* (Figure 6A) the 3 month-lagged pattern of association with CL cases was mainly linear, but for *Lu trapidoi* (Figure 6B) the number of cases seemed to flatten out at high SF abundances. By contrast, monthly CL cases and *Lu panamensis* abundance were negatively and significantly associated with a one and a two month lag (Figure 5SC). As expected from the CCF, the association between CL cases and the 3 month-lagged abundance of *Lu panamensis* had no clear pattern (Figure 6C). When the abundance of *Lu gomezi* and *Lu trapidoi* were added together the maximum positive association occurred at three months of lag, but the positive association was also significant at 2 and 4 months of lag (Figure 5SD). The 3 month-lagged pattern of association between CL cases and the combined abundance of *Lu gomezi* and *Lu trapidoi* (Figure 6D) resembled the one observed for *Lu trapidoi* alone (Figure 6B) with the number of cases flattening out at high SF abundance.

## Discussion

A general criticism for studies addressing the impact of climate change on vector-borne disease transmission is that little to no attention has been given to what changes, if any, occur in entomological risk patterns [66], e.g., what happens to the vectors during ENSO?. Here, we tried to address that knowledge gap for CL by going beyond the description of the association between ENSO and weather patterns and CL epidemics in Panamá, we inquired whether SF vectors change their abundance during ENSO. Our data showed that interannual cycles of CL transmission, as inferred from a CL case time series from Panamá, were associated with ENSO, a pattern observed in neighboring Costa Rica [29], and also observed for malaria in the República de Panamá [67], highlighting the impacts of ENSO on vector-borne diseases in Central America. Large CL Epidemics were observed during the cold ENSO phase or shortly after it, where the delay might reflect the delay between transmission and clinical symptoms in American CL [29], [43], [68], a possibility further re-inforced by the 3 month delayed association between vector abundance and CL incidence.

Seasonal (intrannual time scale) changes in CL transmission were associated with temperature, a weather component with low variability, i.e., low amplitude fluctuations, in the Panamá isthmus. This pattern may make sense in light of Schmalhausen's law, the notion that biological systems are more sensitive to small changes in low variability factors when stressed by other environmental components [65]. SF population dynamics may become more sensitive to changes in temperature given their need to cope with more marked changes in other weather factors, e.g., rainfall which has a more marked seasonal imprint than temperature in Panamá [41]. Indeed, the pattern of higher sensitivity to changes in temperature in places with marked seasonality in rainfall has been observed for other disease vectors [69]. Here, we want to also note that large CL epidemics occurred during or shortly after the cold ENSO phase, a time when, on average, SF vector abundance is the smallest across ENSO phases. Nevertheless, as observed in the raw data, the cold ENSO phase is a time when SF vectors are also prone to show extremely large abundance records per trap-night, which might reflect insect population outbreaks [70], [71], i.e., sudden extraordinary increases in vector abundance [72]. Thus, the occurrence of large CL epidemics during or shortly after the cold ENSO phase might indicate a role for SF vector outbreaks on CL epidemics. Indeed, a detailed study in Venezuelan village showed that CL cases in an endemic village were associated with vector abundance [63], [64]. Nevertheless, the abundance of SF vectors in those studies didn't show potential “outbreaks” [63], [64] in SF abundance, thus not allowing to assess whether CL case incidence flattens out with large vector abundance. This information is necessary to properly understand the role of climate on the entomological risk for CL transmission [29], [62]. This goal will require new longitudinal studies on vector abundance in Panamá [30] where *Leishmania* spp infection in the vectors is also tracked [40], in order to better understand the relationship between vector abundance and vector infection, since constant or nearly constant infections rates in vectors have different implications to understand the role of vectors on transmission patterns. For example, if infection rate decreases with vector abundance, such a density-dependent pattern might partially explain the flattening relationship between vector abundance and cases, such as observed with bloodfeeding success by SF vectors, which decreases with density [73]. Nevertheless, the flattening can also emerge by, or in synergy with, the regulation in the recruitment of susceptible hosts [29] and/or the zoonotic reservoirs, some of which might also experience population outbreaks with ENSO [74], [75] which can ultimately be linked to ENSO mediated changes in the resources sustaining wildlife reservoir populations in the neotropics [76], [77]. Indeed, the most studied wildlife spp reservoir in Panamá, the two toed sloth, has shown relatively large interannual fluctuations in *Leishmania* (*Viannia*) sp. infection [10].

Our results also support the major role of *Lu gomezi* and *Lu trapidoi* as dominant SF vectors of CL in Panamá [2], [43], given their ubiquity across domestic, peridomestic and forest environments. This ubiquity has implications for the role of these species in both the transmission to humans and as bridge of pathogens across vertebrate *Leishmania* spp hosts and eco-epidemiological environments [78]. By contrast, *Lu panamensis* was mainly present in forest environments, which suggests that it might not be heavily involved in domiciliary/peridomiciliary CL transmission, a possibility also put forward by recent studies on spatial patterns of human infections [43] and dogs (unpublished data), which were mainly associated, respectively, with *Lu gomezi* and *Lu trapidoi* abundance, but not *Lu panamensis*. Nevertheless, our inferences are limited given our focus on acknowledged dominant vectors species, which is an approach that potentially biases the identification of other vectors present at CL transmission foci, a problem that can only be solved when studying the whole SF community [79].

Finally, our study has some limitations related to the nature of the data and its countrywide geographical scale. Although our CL and SF data have a consistent quality, there is ample room to improve CL and SF surveillance. For CL surveillance, an urgent need is to standardize diagnostics across the country using sensitive and specific methods [1], [26]. Even if it is impossible to standardize diagnostics at the health post level, an effort should be made to estimate the error in diagnostics, as done for malaria, where all clinically diagnosed cases are confirmed at the ICGES, and quality controls on the specificity of diagnosis are equally performed [67]. The non-spatial nature of our analysis precludes the identification of transmission hotspots requiring attention [80] or zones were biases in case report might be occurring [81]. Similarly, the role that patterns of socio-economic inequity might have in the impacts of climate change and weather variability on CL transmission [32] cannot be estimated. Nevertheless, the relative low variability in rainfall patterns across human inhabited zones in Panamá [41] suggests that a country-wise analysis is a sound method to make inferences about the relationship between CL transmission, ENSO and weather patterns. The CL cases time series showed no trend which allowed us to ignore a denominator for the cases, nevertheless we cannot assert whether the lack of trends is due to stationary population patterns in the population at risk, or if they reflect other unknown changes in the populations at risk and/or transmission. This point could be further clarified by the establishment of health demographic surveillance systems that could both improve the understanding of disease transmission patterns and the demography of populations living in CL endemic areas. Similarly, SF monitoring can be improved and more systematically done at endemic areas. This is an issue of major importance, since, given the delay between transmission and clinical CL, an early prediction of CL epidemics will be more robust if based on the monitoring of SF abundance and *Leishmania* sp. infection [29]. Although each SF abundance estimate came, on average, from ten trap-nights, locations were variable and in some instances SF estimates came from as few as three tree-trap nights and one location, yet these scarce records are abundant in the context of entomological surveillance for CL, and for most neglected tropical diseases. In that sense, an effort could be made to establish sentinel posts in highly endemic counties, thus rendering feasible a highly standardized estimation of SF abundance across endemic areas, where ideally vector infection is also tracked and this information used for prediction and pro-active vector control [31].

## Conclusion

Our data clearly supports that changes in SF abundance and CL cases reported at health facilities in Panamá are associated with ENSO. Interannual variability in CL cases is associated with ENSO, where large epidemics follow the cold ENSO phase, while seasonal patterns are associated with temperature and rainfall variability. CL cases were positively associated with 3-month lagged *Lu gomezi* and *Lu trapidoi* abundance estimates from light traps. SF vector abundance, on average, decreased during the hot and cold ENSO phases, when compared with the normal ENSO phase, yet variability in SF was largest during the cold ENSO phase suggesting that SF population outbreaks might play a role in CL epidemics, a subject deserving further research.

## Supporting Information

Figure S1 **Sand fly sampling locations in the República de Panamá.** (A) República de Panamá location in the neotropics (B) Species composition at each sampling point (C) Eco-epidemiological sampling environments at each location (D) Sampling year. In each panel the legend indicates the color coding for points.
(PDF)

Figure S2 **Boxplots of monthly Cutaneous Leishmaniasis cases as function of El Niño Southern Oscillation (ENSO) phase.** Boxes contain data within the 25th to 75th quantiles. Lines inside the boxes show the median of the distribution for each month.
(PDF)

Figure S3 **Cutaneous leishmaniasis cases and climatic covariates correlation functions.** (A) Cutaneous Leishmaniasis cases, Leish, from Republic of Panamá partial autocorrelation function, PACF. Cross-Correlation Functions, CCFs, between Leish and (B) Sea Surface Temperature 4, i.e., El Niño 4 Index (C) Rainfall (D) Maximum Temperature (E) Minimum Temperature and (F) Average Temperature. Blue dashed lines indicate the 95% confidence limits for correlations that can be expected by random.
(PDF)

Figure S4 **Cutaneous Leishmaniasis cases from Republic of Panamá autocorrelation function (ACF).** The ACF is based on monthly data from January 2000 to December 2010. Blue dashed lines indicate the 95% confidence limits for correlations that can be expected by random.
(PDF)

Figure S5 **Cutaneous Leishmaniasis cases and Sand Fly vector abundance Cross Correlation Functions (CCFs).** CCFs between the number of monthly CL cases and sand fly vector abundance/trap night/month: (A) *Lutzomyia gomezi* (B) *Lutzomyia trapidoi* (C) *Lutzomyia panamensis* (D) *Lutzomyia gomezi* and *Lutzomyia trapidoi*. Blue dashed lines indicate the 95% confidence limits for correlations that can be expected by random.
(PDF)

Table S1 **Cutaneous Leishmaniasis time series model selection.** Columns indicate the type of model (models): Null, full or the backward elimination round, the autoregressive (Autoregressive) and seasonal (Seasonal) order, the covariates, including inside parenthesis the lag (Covariates(Lag)) and The best model for the null and full models and each selection round are **bolded**. o and x indicate, respectively, the presence or absence of a variable in a model. SST4, Temp and Rain are, respectively, abbreviations for Sea Surface Temperature 4 (El Niño 4 Index), Temperature and Rainfall.
(PDF)

Table S2 **Sand Fly vector species abundance model selection.** AIC and BIC stand, respectively, for Akaike and Bayesian Information Criteria. Best model selection is guided by their minimization. Best models are **bolded**.
(PDF)

## References

[1] Alvar J, Vélez ID, Bern C, Herrero M, Desjeux P, et al (2012) Leishmaniasis Worldwide and Global Estimates of Its Incidence. PLoS ONE 7: e35671. 22693548 10.1371/journal.pone.0035671 PMC3365071

[2] Christensen HA, Fairchild GB, Herrer A, Johnson CM, Young DG, et al (1983) The ecology of cutaneous leishmaniasis in the republic of Panama. Journal of Medical Entomology 20: 463–484. 6358496 10.1093/jmedent/20.5.463

[3] Garnham PCC (1965) The Leishmanias, with Special Reference to the Role of Animal Reservoirs. American Zoologist 5: 141–151. 14281919 10.1093/icb/5.1.141

[4] Chaves LF, Hernandez M-J, Dobson AP, Pascual M (2007) Sources and sinks: revisiting the criteria for identifying reservoirs for American cutaneous leishmaniasis. Trends in Parasitology 23: 311–316. 17524806 10.1016/j.pt.2007.05.003

[5] Christensen HA, de Vasquez AM, Petersen JL (1999) Short report epidemiologic studies on cutaneous leishmaniasis in eastern Panama. The American Journal of Tropical Medicine and Hygiene 60: 54–57. 9988322 10.4269/ajtmh.1999.60.54

[6] Herrer A, Christensen HA (1976) Epidemiological Patterns of Cutaneous Leishmaniasis in Panama: III. Endemic Persistence of the Disease. The American Journal of Tropical Medicine and Hygiene 25: 54–58. 816214 10.4269/ajtmh.1976.25.54

[7] Dutari L, Loaiza J (2014) American cutaneous leishmaniasis in Panama: a historical review of entomological studies on anthropophilic *Lutzomyia* sand fly species. Parasites & Vectors 7: 218. 24886629 10.1186/1756-3305-7-218 PMC4026118

[8] Christensen HA, de Vasquez AM (1982) The Tree-Buttress Biotope: a Pathobiocenose of *Leishmania braziliensis* . The American Journal of Tropical Medicine and Hygiene 31: 243–251. 7072887 10.4269/ajtmh.1982.31.243

[9] Herrer A, Telford SR Jr (1969) *Leishmania braziliensis* Isolated from Sloths in Panama. Science 164: 1419–1420. 5783717 10.1126/science.164.3886.1419

[10] Herrer A, Christensen HA (1980) *Leishmania braziliensis* in the Panamanian Two-Toed Sloth, *Choloepus hoffmanni* . The American Journal of Tropical Medicine and Hygiene 29: 1196–1200. 7446811 10.4269/ajtmh.1980.29.1196

[11] Herrer A, Telford SR Jr, Christensen HA (1971) Enzootic cutaneous leishmaniasis in Eastern Panama. I. Investigation of the infection among forest mammals. Annals of Tropical Medicine and Parasitology 65: 349–358. 4999520 10.1080/00034983.1971.11686764

[12] Telford SR Jr, Herrer A, Christensen HA (1972) Enzootic cutaneous leishmaniasis in eastern Panama. 3. Ecological factors relating to the mammalian hosts. Annals of Tropical Medicine and Parasitology 66: 173–179. 5038242 10.1080/00034983.1972.11686813

[13] Tesh RB, Chaniotis BN, Aronson MD, Johnson KM (1971) Natural Host Preferences of Panamanian Phlebotomine Sandflies as Determined by Precipitin Test. The American Journal of Tropical Medicine and Hygiene 20: 150–156. 5567741 10.4269/ajtmh.1971.20.150

[14] Tesh RB, Chaniotis BN, Carrera BR, Johnson KM (1972) Further studies on the natural host preferences of Panamanian phlebotomine sandflies. American Journal of Epidemiology 95: 88–93. 5007366 10.1093/oxfordjournals.aje.a121374

[15] Chaniotis BN, Correa MA, Tesh RB, Johnson KM (1974) Horizontal and vertical movements of phlebotomine sandflies in a Panamanian rain forest. Journal of Medical Entomology 11: 369–375. 4852289 10.1093/jmedent/11.3.369

[16] Hanson WJ (1961) The Breeding Places of Phlebotomus in Panama (Diptera, Psychodidae). Annals of the Entomological Society of America 54: 317–322.

[17] Rutledge LC, Mosser HL (1972) Biology of Immature Sandflies (Diptera: Psychodidae) at the Bases of Trees in Panama. Environ Entomol 1: 300–309.

[18] Rutledge LC, Ellenwood DA (1975) Production of Phlebotomine Sandflies on the Open Forest Floor in Panama: The Species Complement. Environ Entomol 4: 71–77.

[19] Rutledge LC, Ellenwood DA (1975) Production of Phlebotomine Sandflies on the Open Forest Floor in Panama: Phytologic and Edaphic Relations. Environ Entomol 4: 83–89.

[20] Rutledge LC, Ellenwood DA (1975) Production of Phlebotomine Sandflies on the Open Forest Floor in Panama: Hydrologic and Physiographic Relations. Environ Entomol 4: 78–82.

[21] Chaniotis BN, Tesh RB, Correa MA, Johnson KM (1972) Diurnal resting sites of phlebotomine sandflies in a Panamanian tropical forest. Journal of Medical Entomology 9: 91–98. 5019215 10.1093/jmedent/9.1.91

[22] Rutledge LG, Ellenwood DA, Johnston L (1975) An analysis of Sand Fly Light trap collections in the Panama canal zone (Diptera: Psychodidae) Journal of Medical Entomology. 12: 179–183. 10.1093/jmedent/12.2.179 1159739

[23] Rutledge LC, Walton BC, Ellenwood DA, Correa MA (1976) A Transect Study of Sand Fly Populations in Panama (Diptera, Psychodidae). Environ Entomol 5: 1149–1154.

[24] Chaniotis BN (1983) Improved trapping of phlebotomine sand flies (Diptera: Psychodidae) in light traps supplemented with dry ice in a neotropical rain forest. Journal of Medical Entomology 20: 222–223. 6405036 10.1093/jmedent/20.2.222

[25] Miranda A, Carrasco R, Paz H, Pascale JM, Samudio F, et al (2009) Molecular Epidemiology of American Tegumentary Leishmaniasis in Panama. The American Journal of Tropical Medicine and Hygiene 81: 565–571. 19815867 10.4269/ajtmh.2009.08-0265

[26] Miranda A, Saldaña A, González K, Paz H, Santamaría G, et al (2012) Evaluation of PCR for cutaneous leishmaniasis diagnosis and species identification using filter paper samples in Panama, Central America. Transactions of the Royal Society of Tropical Medicine and Hygiene 106: 544–548. 22818741 10.1016/j.trstmh.2012.05.005

[27] Chaves L, Calzada J, Rigg C, Valderrama A, Gottdenker N, et al (2013) Leishmaniasis sand fly vector density reduction is less marked in destitute housing after insecticide thermal fogging. Parasites & Vectors 6: 164. 23742709 10.1186/1756-3305-6-164 PMC3693930

[28] Chaves LF, Pascual M (2006) Climate cycles and forecasts of cutaneous leishmaniasis, a nonstationary vector-borne disease. PLos Med 3: 1320–1328. 10.1371/journal.pmed.0030295 PMC1539092 16903778

[29] Chaves LF (2009) Climate and recruitment limitation of hosts: the dynamics of American cutaneous leishmaniasis seen through semi-mechanistic seasonal models. Annals of Tropical Medicine and Parasitology 103: 221–234. 19341537 10.1179/136485909X398267

[30] Chaniotis BN, Neely JM, Correa MA, Tesh RB, Johnson KM (1971) Natural population dynamics of Phlebotomine Sandflies in Panama. Journal of Medical Entomology 8: 339–352. 4334027 10.1093/jmedent/8.4.339

[31] Chaves LF, Pascual M (2007) Comparing Models for Early Warning Systems of Neglected Tropical Diseases. PLoS Negl Trop Dis 1: e33. 17989780 10.1371/journal.pntd.0000033 PMC2041810

[32] Chaves LF, Cohen JM, Pascual M, Wilson ML (2008) Social Exclusion Modifies Climate and Deforestation Impacts on a Vector-Borne Disease. PLoS Neglected Tropical Diseases 2: e176. 18265876 10.1371/journal.pntd.0000176 PMC2238711

[33] Salomon OD, Quintana MG, Mastrángelo AD, Fernandez MS (2012) Leishmaniasis and Climate Change-Case Study: Argentina. Journal of Tropical Medicine 2012: 601242. 22685477 10.1155/2012/601242 PMC3364011

[34] Condino MLF, Galati EAB, Holcman MM, Salum MRB, Silva DCd, et al (2008) Leishmaniose tegumentar americana no Litoral Norte Paulista, período 1993 a 2005. Revista da Sociedade Brasileira de Medicina Tropical 41: 635–641. 19142444 10.1590/s0037-86822008000600015

[35] Rodríguez E-M, Díaz F, Pérez M-V (2013) Spatio-temporal clustering of American Cutaneous Leishmaniasis in a rural municipality of Venezuela. Epidemics 5: 11–19. 23438427 10.1016/j.epidem.2012.10.002

[36] Roger A, Nacher M, Hanf M, Drogoul AS, Adenis A, et al (2013) Climate and Leishmaniasis in French Guiana. The American Journal of Tropical Medicine and Hygiene 89: 564–569. 23939706 10.4269/ajtmh.12-0771 PMC3771301

[37] Confalonieri UEC, Margonari C, Quintão AF (2014) Environmental change and the dynamics of parasitic diseases in the Amazon. Acta Tropica 129: 33–41. 24056199 10.1016/j.actatropica.2013.09.013

[38] Alessi CÁC, Galati EAB, Alves JR, Corbett CEP (2009) American cutaneous leishmaniasis in the Pontal of Paranapanema - SP, Brazil: ecological and entomological aspects. Revista do Instituto de Medicina Tropical de São Paulo 51: 277–282. 19893981 10.1590/s0036-46652009000500008

[39] Toumi A, Chlif S, Bettaieb J, Alaya NB, Boukthir A, et al (2012) Temporal Dynamics and Impact of Climate Factors on the Incidence of Zoonotic Cutaneous Leishmaniasis in Central Tunisia. PLoS Negl Trop Dis 6: e1633. 22563513 10.1371/journal.pntd.0001633 PMC3341328

[40] Anderson JM, Samake S, Jaramillo-Gutierrez G, Sissoko I, Coulibaly CA, et al (2011) Seasonality and Prevalence of *Leishmania major* Infection in *Phlebotomus duboscqi* Neveu-Lemaire from Two Neighboring Villages in Central Mali. PLoS Negl Trop Dis 5: e1139. 21572984 10.1371/journal.pntd.0001139 PMC3091838

[41] Autoridad Nacional del Ambiente (2010) Atlas Ambiental de la República de Panamá. Ciudad de Panamá: Editora Novo Art. 187 p.

[42] Wijeyaratne PM, Arsenault LKJ, Murphy CJ (1994) Endemic disease and development: the leishmaniases. Acta Tropica 56: 349–364. 8023758 10.1016/0001-706x(94)90106-6

[43] Saldaña A, Chaves LF, Rigg CA, Wald C, Smucker JE, et al (2013) Clinical Cutaneous Leishmaniasis Rates Are Associated with Household *Lutzomyia gomezi*, *Lu. panamensis*, and *Lu. trapidoi* Abundance in Trinidad de Las Minas, Western Panama. The American Journal of Tropical Medicine and Hygiene 88: 572–574. 23339202 10.4269/ajtmh.12-0579 PMC3592543

[44] Montenegro J (1926) A cutis-reação na leishmaniose. An Fac Med Univ São Paulo 1: 323–330.

[45] Barry MA, Koshelev MV, Sun GS, Grekin SJ, Stager CE, et al (2014) Cutaneous Leishmaniasis in Cuban Immigrants to Texas who Traveled through the Darién Jungle, Panama. The American Journal of Tropical Medicine and Hygiene 91: 345–347. 24865687 10.4269/ajtmh.14-0124 PMC4125260

[46] Cannella AP, Nguyen BM, Piggott CD, Lee RA, Vinetz JM, et al (2011) A Cluster of Cutaneous Leishmaniasis Associated with Human Smuggling. The American Journal of Tropical Medicine and Hygiene 84: 847–850. 21633017 10.4269/ajtmh.2011.10-0693 PMC3110366

[47] Smith TM, Reynolds RW, Peterson TC, Lawrimore J (2008) Improvements to NOAA's Historical Merged Land–Ocean Surface Temperature Analysis (1880–2006). Journal of Climate 21.

[48] Azpurua J, De La Cruz D, Valderama A, Windsor D (2010) *Lutzomyia* Sand Fly Diversity and Rates of Infection by *Wolbachia* and an Exotic *Leishmania* Species on Barro Colorado Island, Panama. PLoS Negl Trop Dis 4: e627. 20231892 10.1371/journal.pntd.0000627 PMC2834748

[49] Espinoza Areas PM (2002) Determinación de la composición de especies y abundancia relativa del género *Lutzomyia* Franca, 1924 (Diptera: Psychodidae) en el distrito de Arraijan, corregimiento de Santa Clara, República de Panamá. Panamá: Universidad de Panamá. 50 p.

[50] Valderrama A, Herrera M, Salazar A (2008) Relación entre la composición de especies del género de *Lutzomyia* Franca (Diptera: Psychodidae, Phlebotominae) y los diferentes tipos de bosques en Panamá. Acta Zoológica Mexicana 24: 67–68.

[51] Young DG, Duncan MA (1994) Guide to the identification and geographic distribution of *Lutzomyia* sand flies in Mexico, the West Indies, Central and South America (Diptera: Psychodidae). Gainesville, FL: Associated Publishers. 881 p.

[52] Venables WN, Ripley BD (2002) Modern applied statistics with S. New York: Springer.

[53] Shumway RH, Stoffer DS (2011) Time series analysis and its applications: New York: Springer. 572 p.

[54] Faraway JJ (2004) Linear Models with R. Boca Raton: CRC Press.

[55] Kuhn M, Johnson K (2013) Applied Predictive Modeling. New York: Springer. 600 p.

[56] Cazelles B, Chavez M, Magny GCd, Guégan J-F, Hales S (2007) Time-dependent spectral analysis of epidemiological time-series with wavelets. Journal of The Royal Society Interface 4: 625–636. 10.1098/rsif.2007.0212 PMC2373388 17301013

[57] Cazelles B, Chavez M, Berteaux D, Ménard F, Vik J, et al (2008) Wavelet analysis of ecological time series. Oecologia 156: 287–304. 18322705 10.1007/s00442-008-0993-2

[58] Bolker BM, Brooks ME, Clark CJ, Geange SW, Poulsen JR, et al (2009) Generalized linear mixed models: a practical guide for ecology and evolution. Trends in Ecology & Evolution 24: 127–135. 19185386 10.1016/j.tree.2008.10.008

[59] Chaves LF (2010) An Entomologist Guide to Demystify Pseudoreplication: Data Analysis of Field Studies With Design Constraints. Journal of Medical Entomology 47: 291–298. 20496574 10.1603/me09250

[60] Faraway JJ (2006) Extending the Linear Model with R: Generalized Linear, Mixed Effects and Nonparametric Regression Models Boca Raton: CRC Press.

[61] Chaves LF, Hernandez M-J (2004) Mathematical modelling of American Cutaneous Leishmaniasis: incidental hosts and threshold conditions for infection persistence. Acta Tropica 92: 245–252. 15533294 10.1016/j.actatropica.2004.08.004

[62] Chaves LF, Hernandez M-J, Ramos S (2008) Simulación de modelos matemáticos como herramienta para el estudio de los reservorios de la Leishmaniasis Cutánea Americana. Divulgaciones matemáticas 16: 125–154.

[63] Feliciangeli DM, Rabinovich J (1998) Abundance of *Lutzomyia ovallesi* but not *Lu. gomezi* (Diptera: Psychodidae) correlated with cutaneous leishmaniasis incidence in north-central Venezuela. Medical and Veterinary Entomology 12: 121–131. 9622364 10.1046/j.1365-2915.1998.00072.x

[64] Rabinovich JE, Feliciangeli MD (2004) Parameters of *Leishmania braziliensis* transmission by indoor *Lutzomyia ovallesi* in Venezuela. The American Journal of Tropical Medicine and Hygiene 70: 373–382. 15100449

[65] Chaves LF, Koenraadt CJM (2010) Climate Change and Highland Malaria: Fresh Air for a Hot Debate. The Quarterly Review of Biology 85: 27–55. 20337259 10.1086/650284

[66] Reiter P (2001) Climate change and mosquito-borne disease. Environ Health Perspect 109: 141–161. 11250812 10.1289/ehp.01109s1141 PMC1240549

[67] Hurtado LA, Cáceres L, Chaves LF, Calzada JE (2014) When climate change couples social neglect: malaria dynamics in Panamá. Emerging Microbes & Infections 3: e27. 10.1038/emi.2014.27 PMC4008768 26038518

[68] Jirmanus L, Glesby MJ, Guimarães LH, Lago E, Rosa ME, et al (2012) Epidemiological and Clinical Changes in American Tegumentary Leishmaniasis in an Area of *Leishmania (Viannia) braziliensis* Transmission Over a 20-Year Period. The American Journal of Tropical Medicine and Hygiene 86: 426–433. 22403312 10.4269/ajtmh.2012.11-0378 PMC3284357

[69] Chaves LF, Morrison AC, Kitron UD, Scott TW (2012) Nonlinear impacts of climatic variability on the density-dependent regulation of an insect vector of disease. Global Change Biology 18: 457–468.

[70] Berryman AA, Stark RW (1985) Assessing the risk of forest insect outbreaks. Zeitschrift für Angewandte Entomologie 99: 199–208.

[71] Berryman A, Barbosa P, Schultz J (1987) The theory and classification of outbreaks. In: Barbosa P, Schultz JC, editors. Insect outbreaks. San Diego: Academic Press. pp. 3–30.

[72] Chaves LF, Scott TW, Morrison AC, Takada T (2014) Hot temperatures can force delayed mosquito outbreaks via sequential changes in *Aedes aegypti* demographic parameters in autocorrelated environments. Acta Tropica 129: 15–24. 23537497 10.1016/j.actatropica.2013.02.025

[73] Kelly DW, Mustafa Z, Dye C (1996) Density-dependent feeding success in a field population of the sandfly, *Lutzomyia longipalpis* . Journal of Animal Ecology 65: 517–527.

[74] Lima M, Keymer JE, Jaksic FM (1999) El Niño Southern Oscillation driven rainfall variability and delayed density dependence cause rodent outbreaks in western South America: linking demography and population dynamics. The American Naturalist 153: 476–491. 10.1086/303191 29578796

[75] Davis S, Calvet E (2005) Fluctuating rodent populations and risk to humans from rodent-borne zoonoses. Vector-Borne & Zoonotic Diseases 5: 305–314. 16417426 10.1089/vbz.2005.5.305

[76] Adler GH (1998) Impacts of resource abundance on populations of a tropical forest rodent. Ecology 79: 242–254.

[77] Wright SJ, Calderón O (2006) Seasonal, El Niño and longer term changes in flower and seed production in a moist tropical forest. Ecology Letters 9: 35–44. 16958866 10.1111/j.1461-0248.2005.00851.x

[78] Chaves LF, Añez N (2004) Species co-occurrence and feeding behavior in sand fly transmission of American cutaneous leishmaniasis in western Venezuela. Acta Tropica 92: 219–224. 15533290 10.1016/j.actatropica.2004.08.001

[79] Calzada JE, Saldaña A, Rigg C, Valderrama A, Romero L, et al (2013) Changes in phlebotomine sand fly species composition following insecticide thermal fogging in a rural setting of western Panamá. PLoS One 8: e53289. 23536748 10.1371/journal.pone.0053289 PMC3541195

[80] Hansson E, Sasa M, Mattisson K, Robles A, Gutiérrez JM (2013) Using Geographical Information Systems to Identify Populations in Need of Improved Accessibility to Antivenom Treatment for Snakebite Envenoming in Costa Rica. PLoS Negl Trop Dis 7: e2009. 23383352 10.1371/journal.pntd.0002009 PMC3561131

[81] Hansson E, Cuadra S, Oudin A, de Jong K, Stroh E, et al (2010) Mapping Snakebite Epidemiology in Nicaragua – Pitfalls and Possible Solutions. PLoS Negl Trop Dis 4: e896. 21124884 10.1371/journal.pntd.0000896 PMC2990701

