# Supplementary figures and images for: Cutaneous Leishmaniasis and Sand Fly Fluctuations Are Associated with El Niño in Panamá
Source: PLoS Negl Trop Dis. 2014 Oct 2;8(10):e3210. doi: 10.1371/journal.pntd.0003210 (PMC4183471; doi:10.1371/journal.pntd.0003210)

A

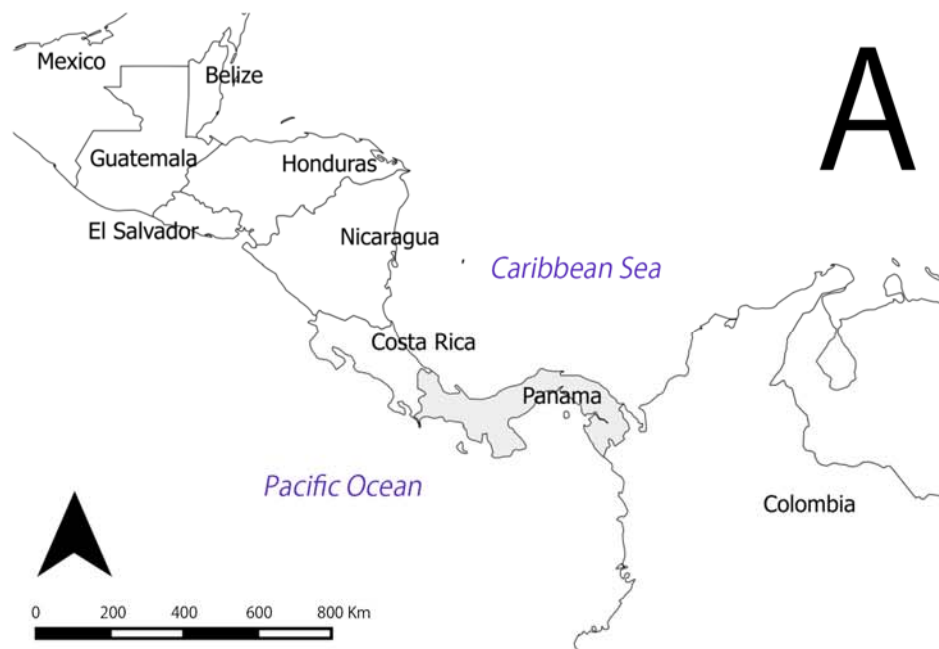

B

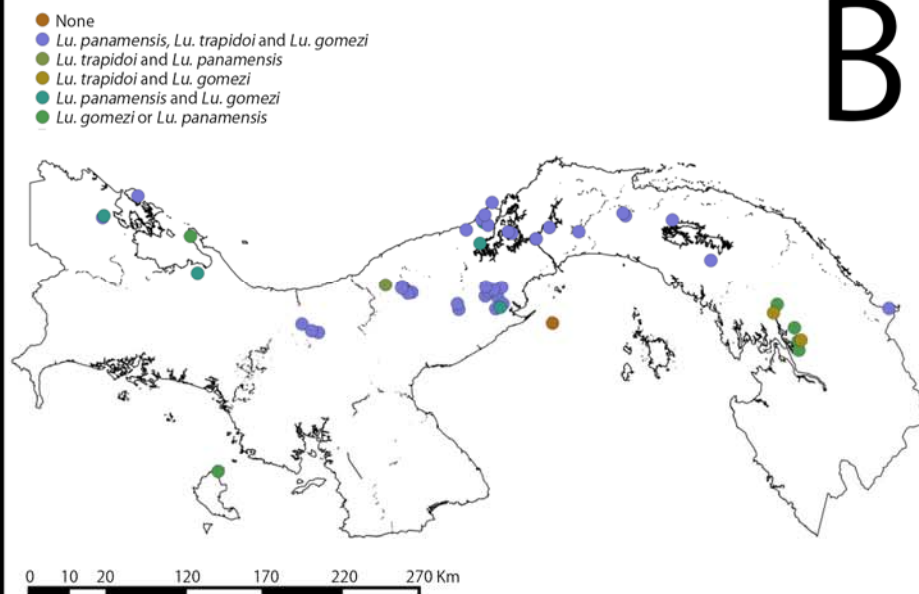

C

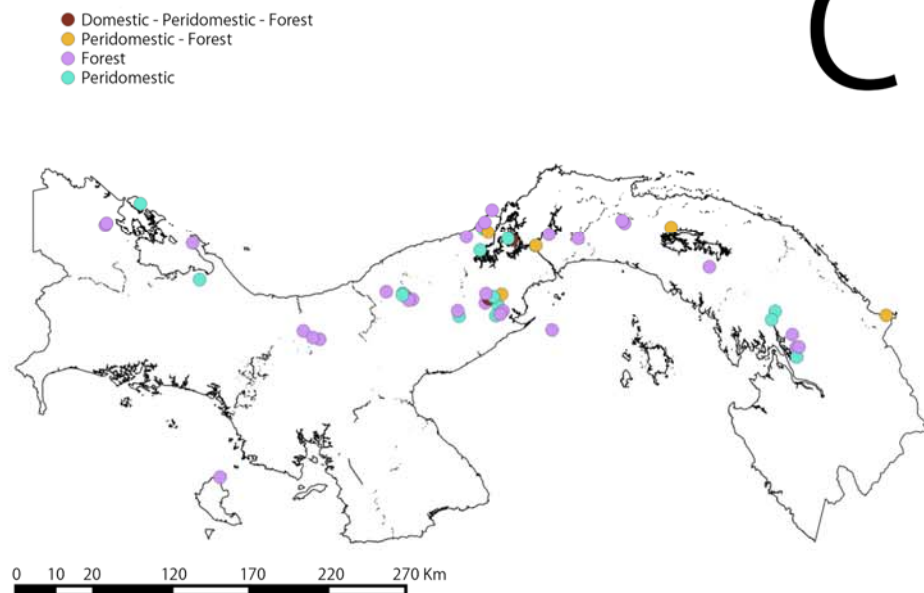

D

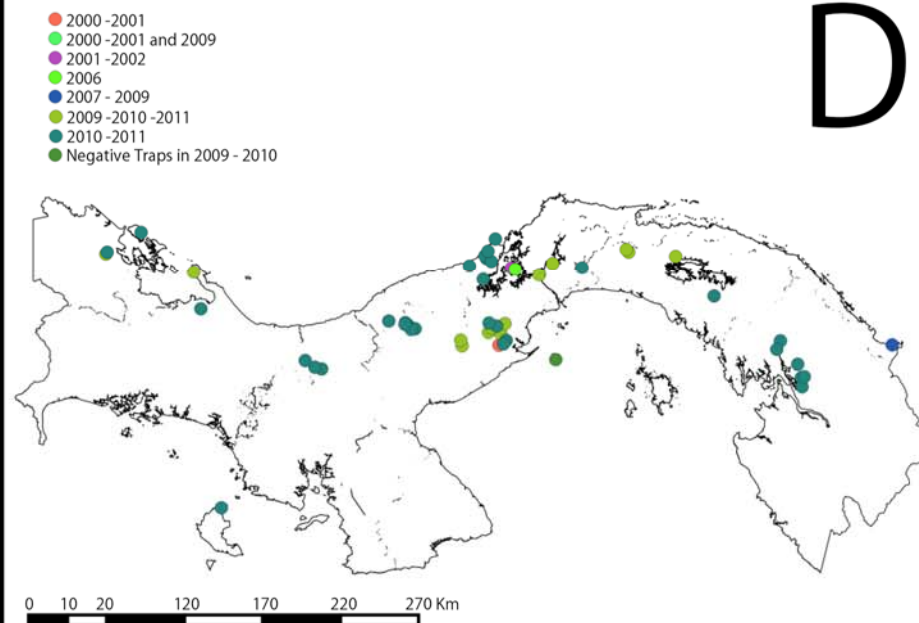

Supplement: Figure S1 — Sand fly sampling locations in the República de Panamá. (A) República de Panamá location in the neotropics (B) Species composition at each sampling point (C) Eco-epidemiological sampling environments at each location (D) Sampling year. In each panel the legend indicates the color coding for points. (PDF) [file pntd.0003210.s001.pdf]

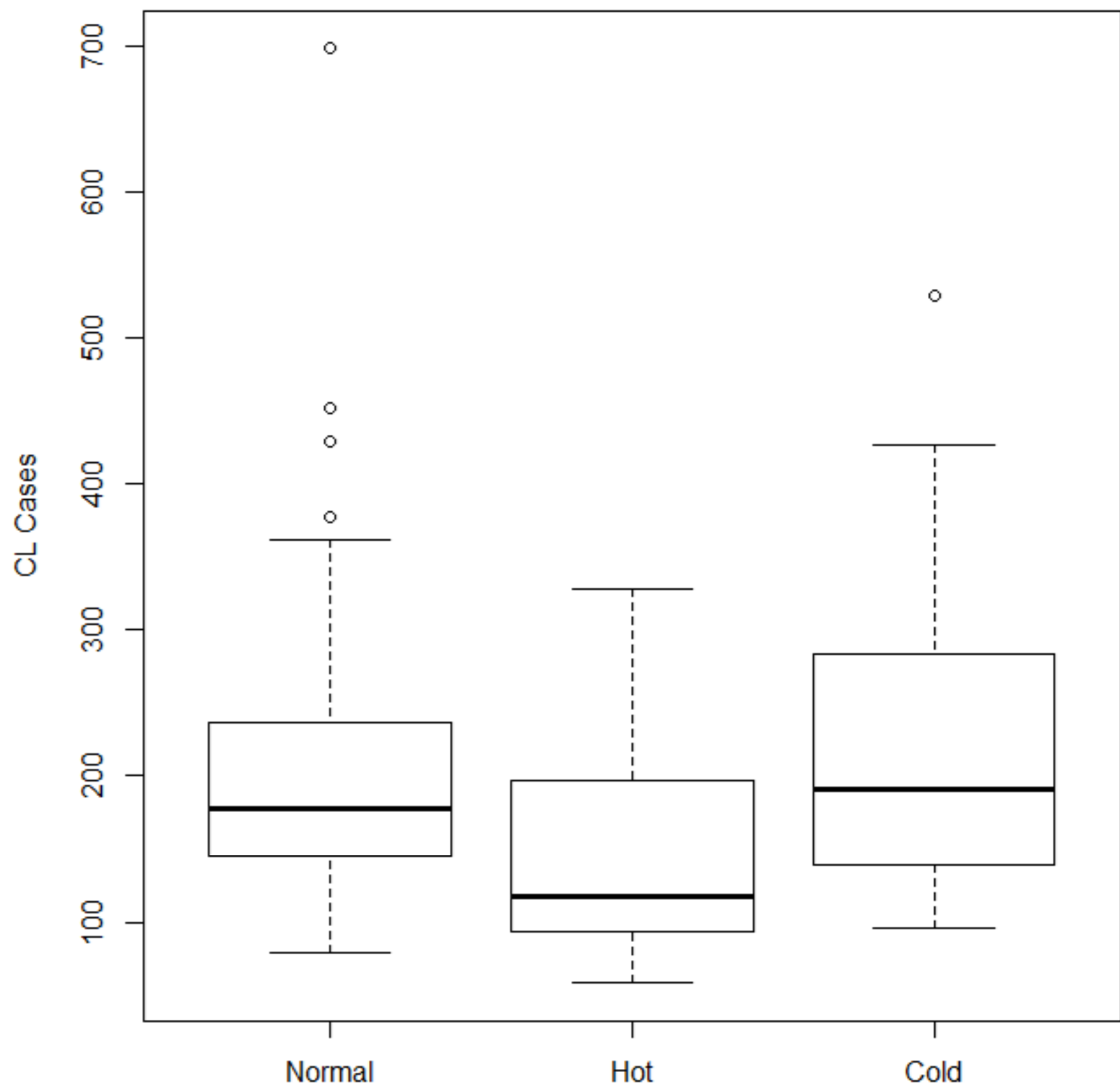

Supplement: Figure S2 — Boxplots of monthly Cutaneous Leishmaniasis cases as function of El Niño Southern Oscillation (ENSO) phase. Boxes contain data within the 25th to 75th quantiles. Lines inside the boxes show the median of the distribution for each month. (PDF) [file pntd.0003210.s002.pdf]

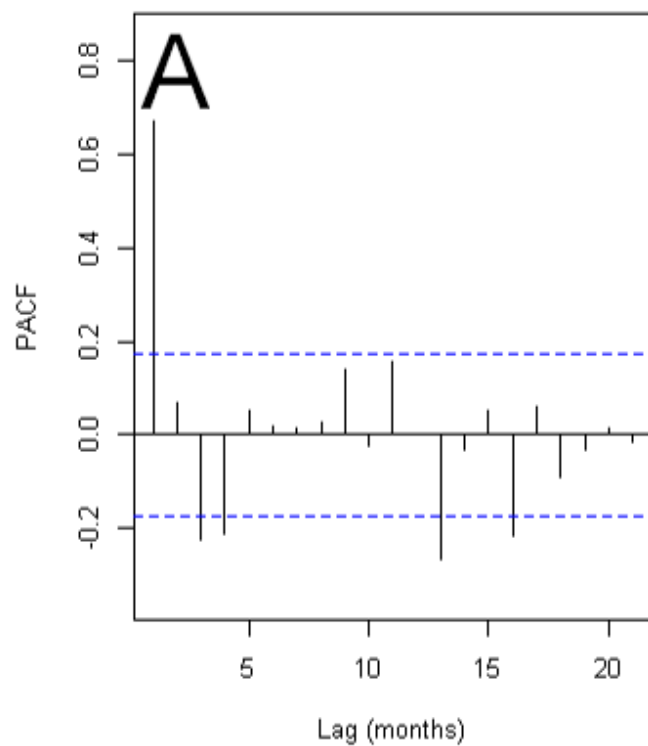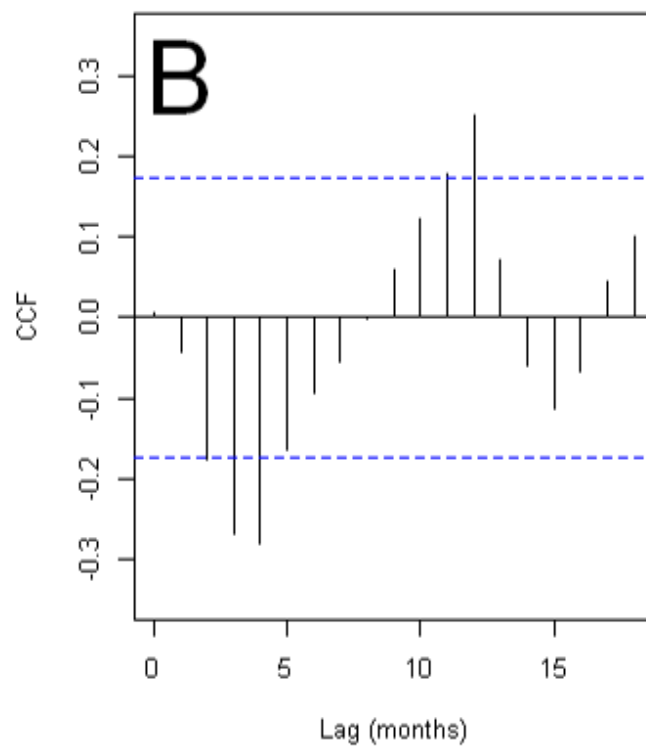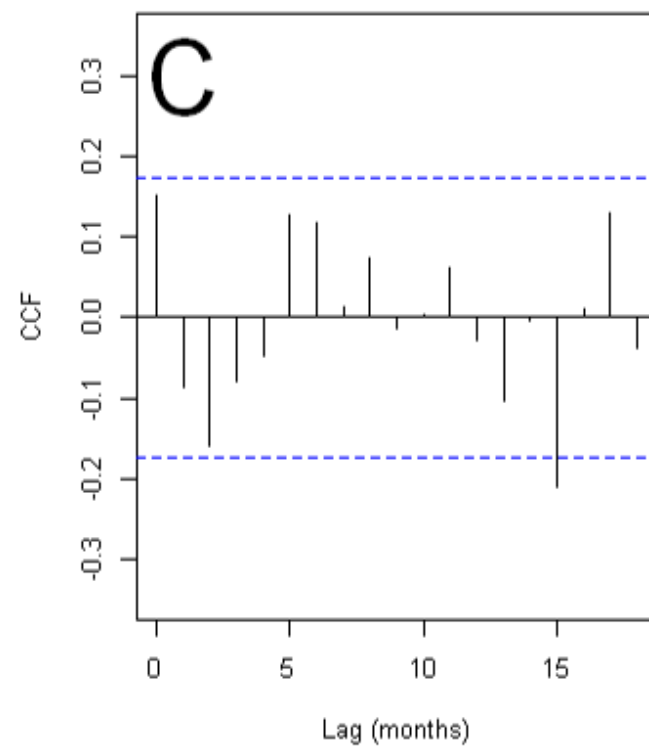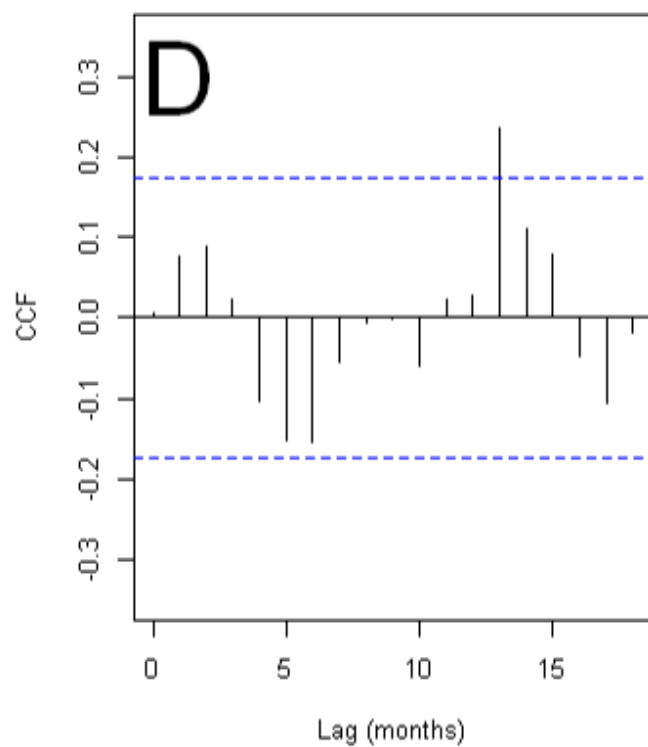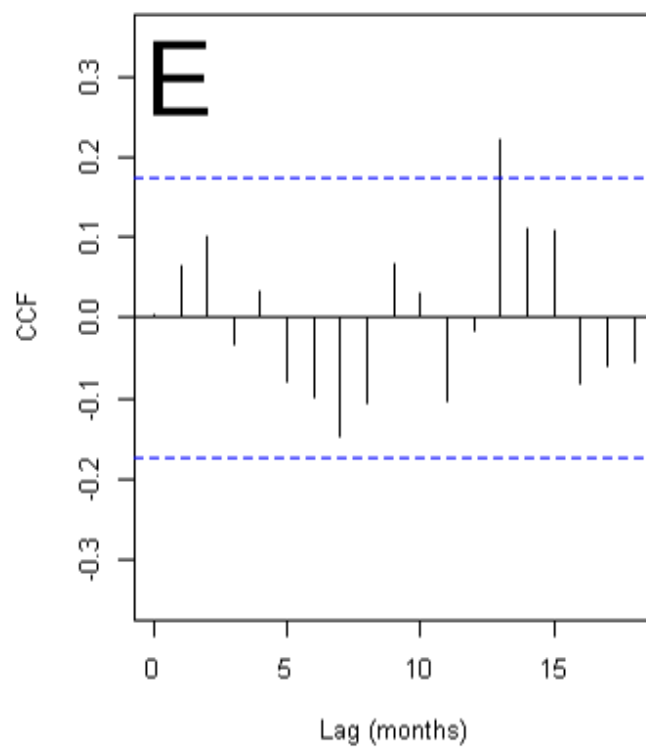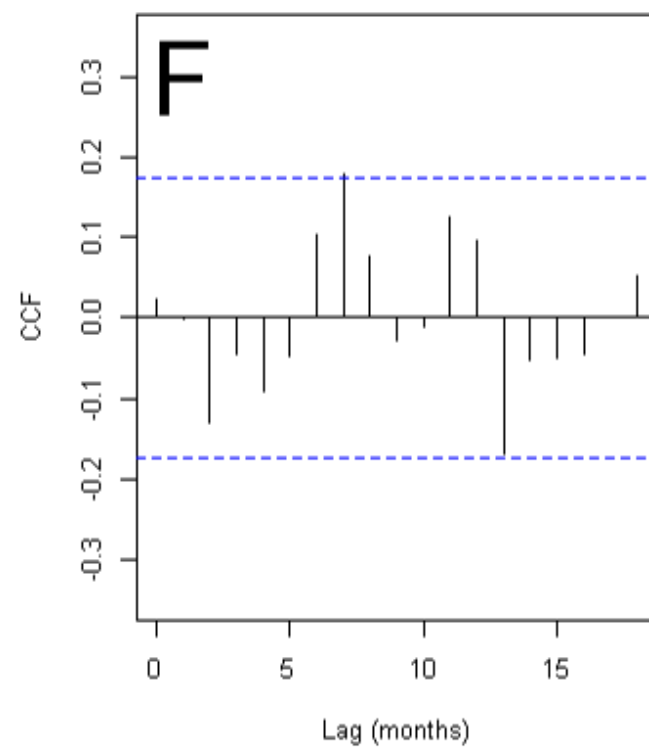

Supplement: Figure S3 — Cutaneous leishmaniasis cases and climatic covariates correlation functions. (A) Cutaneous Leishmaniasis cases, Leish, from Republic of Panamá partial autocorrelation function, PACF. Cross-Correlation Functions, CCFs, between Leish and (B) Sea Surface Temperature 4, i.e., El Niño 4 Index (C) Rainfall (D) Maximum Temperature (E) Minimum Temperature and (F) Average Temperature. Blue dashed lines indicate the 95% confidence limits for correlations that can be expected by random. (PDF) [file pntd.0003210.s003.pdf]

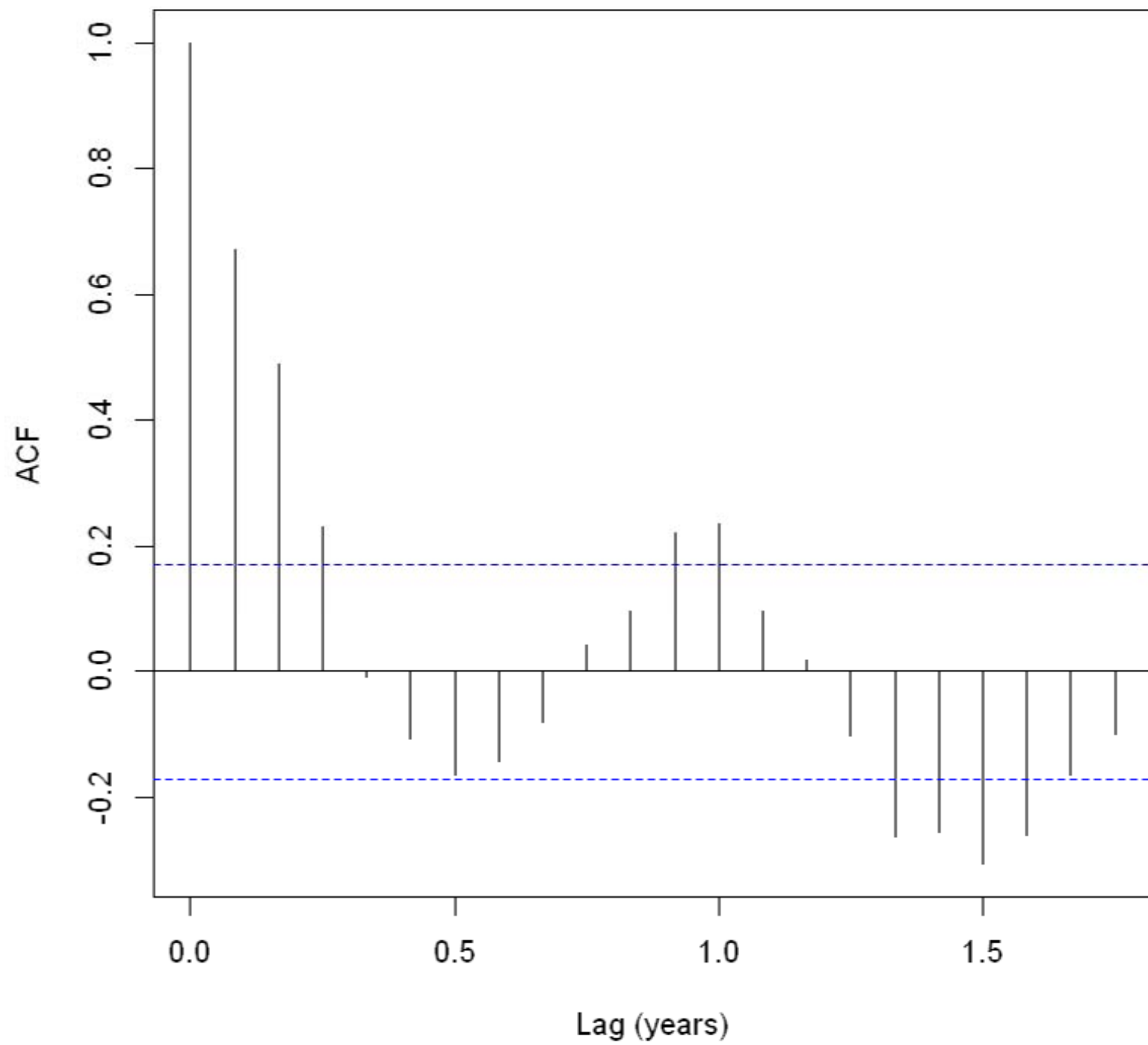

Supplement: Figure S4 — Cutaneous Leishmaniasis cases from Republic of Panamá autocorrelation function (ACF). The ACF is based on monthly data from January 2000 to December 2010. Blue dashed lines indicate the 95% confidence limits for correlations that can be expected by random. (PDF) [file pntd.0003210.s004.pdf]

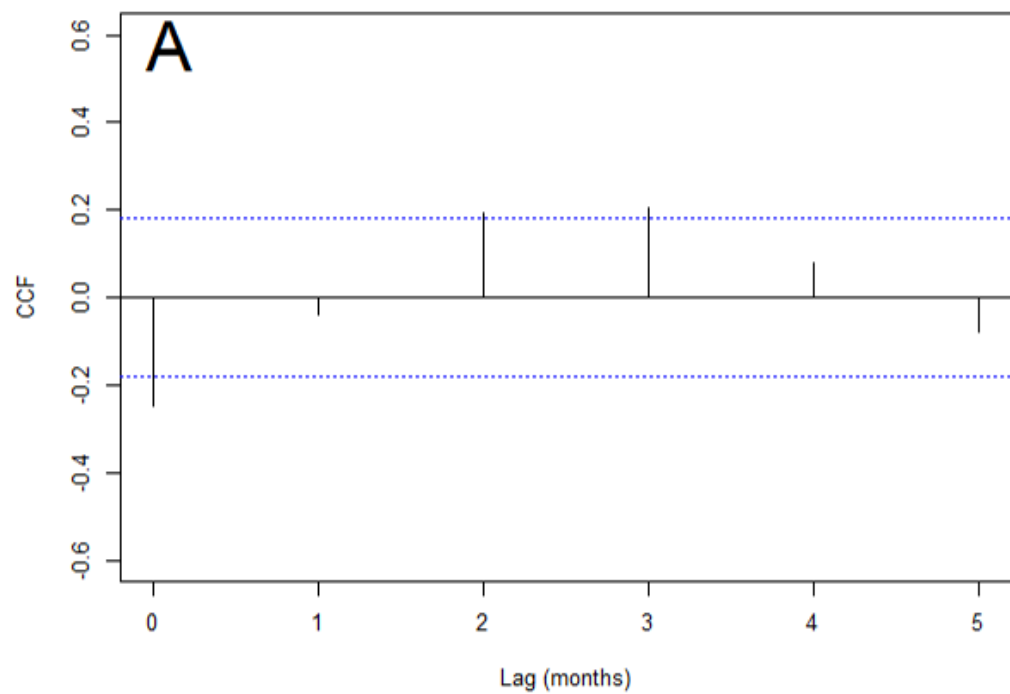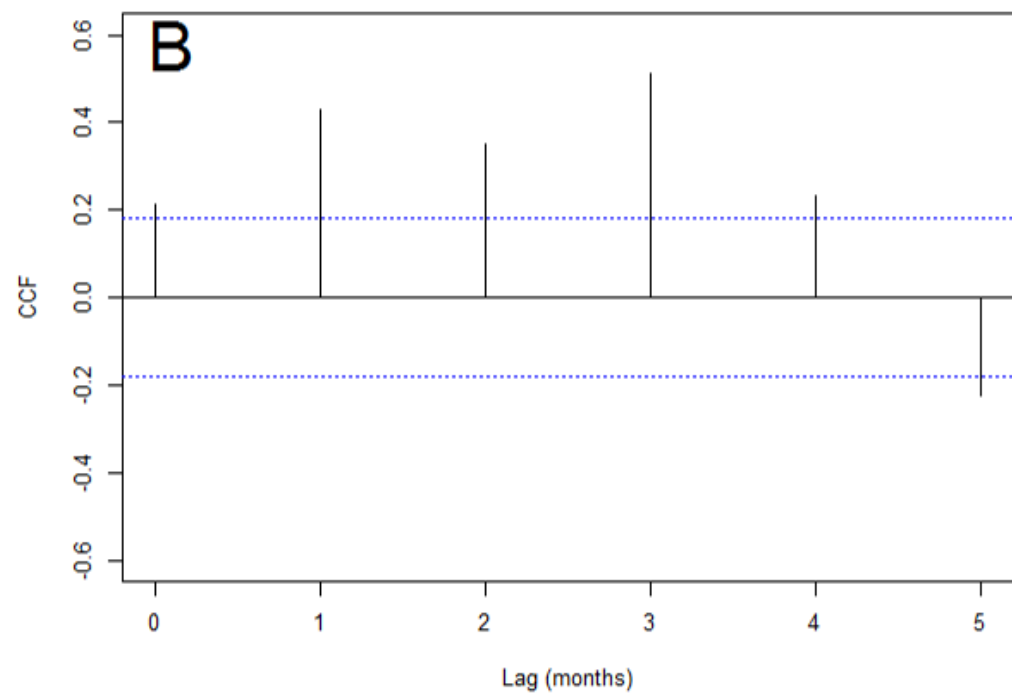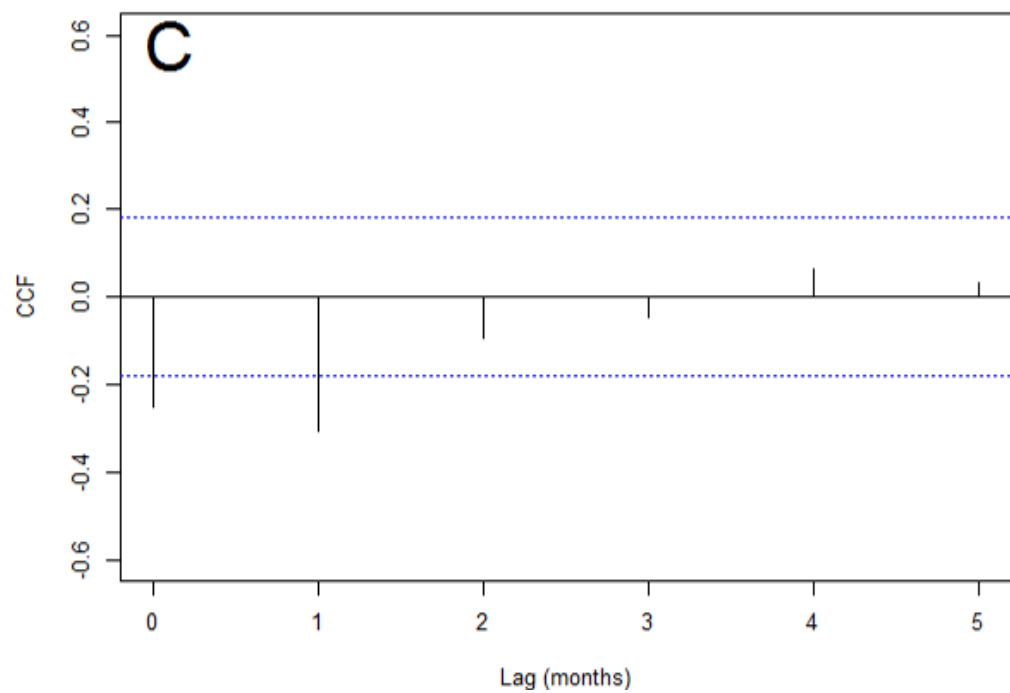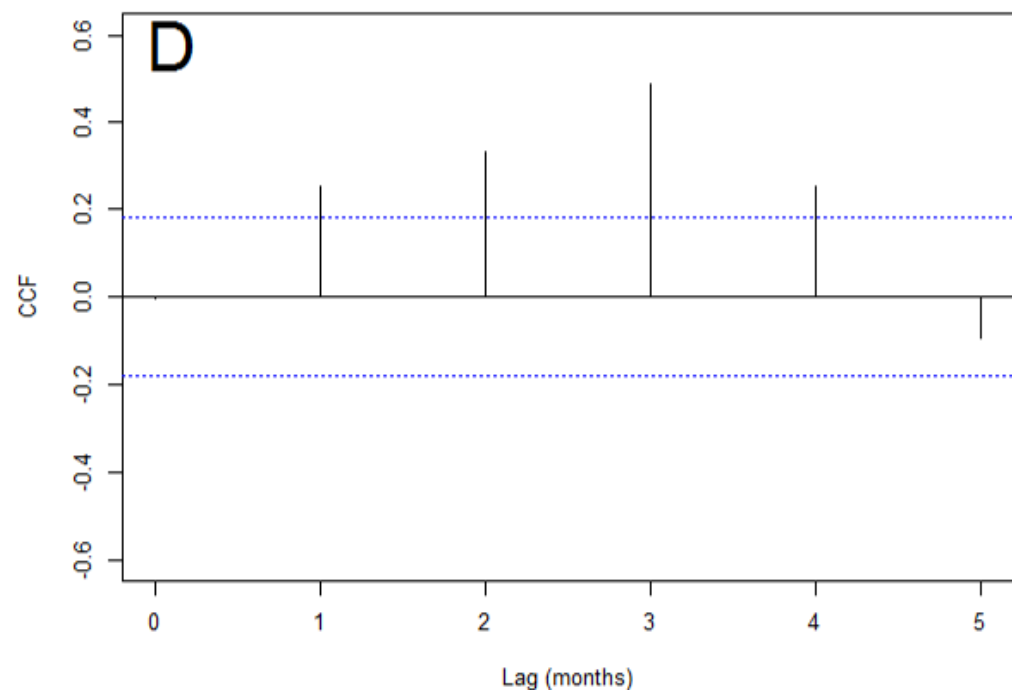

Supplement: Figure S5 — Cutaneous Leishmaniasis cases and Sand Fly vector abundance Cross Correlation Functions (CCFs). CCFs between the number of monthly CL cases and sand fly vector abundance/trap night/month: (A) Lutzomyia gomezi (B) Lutzomyia trapidoi (C) Lutzomyia panamensis (D) Lutzomyia gomezi and Lutzomyia trapidoi. Blue dashed lines indicate the 95% confidence limits for correlations that can be expected by random. (PDF) [file pntd.0003210.s005.pdf]
