# Supplementary material for: Cutaneous Leishmaniasis and Sand Fly Fluctuations Are Associated with El Niño in Panamá
Source: PLoS Negl Trop Dis. 2014 Oct 2;8(10):e3210. doi: 10.1371/journal.pntd.0003210 (PMC4183471; doi:10.1371/journal.pntd.0003210)
Supplement: Table S1 — Cutaneous Leishmaniasis time series model selection. Columns indicate the type of model (models): Null, full or the backward elimination round, the autoregressive (Autoregressive) and seasonal (Seasonal) order, the covariates, including inside parenthesis the lag (Covariates(Lag)) and The best model for the null and full models and each selection round are bolded. o and x indicate, respectively, the presence or absence of a variable in a model. SST4, Temp and Rain are, respectively, abbreviations for Sea Surface Temperature 4 (El Niño 4 Index), Temperature and Rainfall. (PDF) [file pntd.0003210.s006.pdf]

Table S1 Model Selection. Columns indicate the type of model (models): Null, full or the backward elimination round, the autoregressive (Autoregressive) and seasonal (Seasonal) order, the covariates, including inside parenthesis the lag (Covariates(Lag)) and the Akaike Information Criterion (AIC), a model selection which is minimized by the best model. The best model for the null and full models and each selection round are **bolded**. o and x indicate, respectively, the presence or absence of a variable in a model. SST4, Temp and Rain are, respectively, abbreviations for Sea Surface Temperature 4 (El Niño 4 Index), Temperature and Rainfall.

Models	Autoregressive			Seasonal	Covariates (Lag)					AIC
	1st	2nd	3rd	12th	SST4(4)	SST4(5)	SST4(12)	Temp(13)	Rain(15)	
Null Models	o	o	o	o	x	x	x	x	x	**1489**
	o	o	x	o	x	x	x	x	x	1494
	o	x	x	o	x	x	x	x	x	1497
Full	o	o	o	o	o	o	o	o	o	**1296**
1st Round	o	o	o	o	o	o	o	o	x	1296
	o	o	o	o	o	o	o	x	o	1305
	o	o	o	o	o	o	x	o	o	1297
	o	o	o	o	o	x	o	o	o	**1294**
	o	o	o	o	x	o	o	o	o	1302
	o	o	o	x	o	o	o	o	o	1296
	o	o	x	o	o	o	o	o	o	1295
	o	x	x	o	o	o	o	o	o	1309
2nd Round	o	o	o	o	o	x	o	o	x	1295
	o	o	o	o	o	x	o	x	o	1303
	o	o	o	o	o	x	x	o	o	1296
	o	o	o	o	x	x	o	o	o	1300
	o	o	o	x	o	x	o	o	o	1296
	o	o	x	o	o	x	o	o	o	**1293**
	o	x	x	o	o	x	o	o	o	1308
3rd Round	o	o	x	o	o	x	o	o	x	**1293**
	o	o	x	o	o	x	o	x	o	1302
	o	o	x	o	o	x	x	o	o	1308
	o	o	x	o	x	x	o	o	o	1294
	o	o	x	x	o	x	o	o	o	1294
	o	x	x	o	o	x	o	o	o	1307
4th Round	o	o	x	o	o	x	o	x	x	1303
	o	o	x	o	o	x	x	o	x	1310
	o	o	x	o	x	x	o	o	x	1297
	o	o	x	x	o	x	o	o	x	**1293**
	o	x	x	o	o	x	o	o	x	1307
