# Supplementary material for: Cutaneous Leishmaniasis and Sand Fly Fluctuations Are Associated with El Niño in Panamá
Source: PLoS Negl Trop Dis. 2014 Oct 2;8(10):e3210. doi: 10.1371/journal.pntd.0003210 (PMC4183471; doi:10.1371/journal.pntd.0003210)
Supplement: Table S2 — Sand Fly vector species abundance model selection. AIC and BIC stand, respectively, for Akaike and Bayesian Information Criteria. Best model selection is guided by their minimization. Best models are bolded. (PDF) [file pntd.0003210.s007.pdf]

Table S2 Sand Fly vector species abundance model selection. AIC and BIC stand, respectively, for Akaike and Bayesian Information Criteria. Best model selection is guided by their minimization. The symbol | indicates the nesting of random factors. Best models are **bolded**.

Sand Fly Vector Species	Random Factors	Fixed Factors	BIC	AIC
Lutzomyia gomezi	Locations|Study, Study, Year	ENSO Phase, sampling month, eco-epidemiological environment	3267.4	3341.7
	Locations, Year	ENSO Phase, sampling month, eco-epidemiological environment	**3265.4**	**3335.8**
Lu. trapidoi	Locations|Study, Study, Year	ENSO Phase, sampling month, eco-epidemiological environment	3547.3	3620.7
	Locations, Year	ENSO Phase, sampling month, eco-epidemiological environment	**3545.3**	**3614.8**
Lu. panamensis	Locations|Study, Study, Year	ENSO Phase, sampling month, eco-epidemiological environment	7748.0	7820.3
	Locations, Year	ENSO Phase, sampling month, eco-epidemiological environment	**7748.0**	**7816.6**
